# Analysis of Microbiome for AP and CRC Discrimination

**DOI:** 10.3390/bioengineering12070713

**Published:** 2025-06-29

**Authors:** Alessio Rotelli, Ali Salman, Leandro Di Gloria, Giulia Nannini, Elena Niccolai, Alessio Luschi, Amedeo Amedei, Ernesto Iadanza

**Affiliations:** 1 Department of Medical Biotechnologies, University of Siena, 53100 Siena, Italy; a.rotelli1@student.unisi.it (A.R.); a.salman@student.unisi.it (A.S.); ernesto.iadanza@unisi.it (E.I.); 2 Department of Clinical and Experimental Medicine, University of Florence, 50134 Florence, Italy; leandro.digloria@unifi.it (L.D.G.); giulia.nannini@unifi.it (G.N.); elena.niccolai@unifi.it (E.N.); amedeo.amedei@unifi.it (A.A.)

**Keywords:** microbiome, synthetic data augmentation, machine learning, adenomatous polyps, colorectal cancer

## Abstract

Microbiome data analysis is essential for understanding the role of microbial communities in human health. However, limited data availability often hinders research progress, and synthetic data generation could offer a promising solution to this problem. This study aims to explore the use of machine learning (ML) to enrich an unbalanced dataset consisting of microbial operational taxonomic unit (OTU) counts of 148 samples, belonging to 61 patients. In detail, 34 samples are from 16 adenomatous polyps (AP) patients, while 114 samples are from 46 colorectal cancer (CRC) patients. Synthesis of AP and CRC samples was conducted using the Synthetic Data Vault Python library, employing a Gaussian Copula synthesiser. Subsequently, the synthesised data quality was evaluated using a logistic regression model in parallel with an optimised support vector machine algorithm (polynomial kernel). The data quality is considered good when neither of the two algorithms can discriminate between real and synthetic data, showing low accuracy, F1 score, and precision values. Furthermore, additional statistical tests were employed to confirm the similarity between real and synthetic data. After data validation, layer-wise relevance propagation (LRP) was performed on a deep learning classifier to extract important OTU features from the generated dataset, to discriminate between CRC patients and those affected by AP. Exploiting the acquired features, which correspond to unique bacterial taxa, ML classifiers were trained and tested to estimate the validity of such microorganisms in recognising AP and CRC samples. The simplified version of the original OTU table opens up opportunities for further investigations, especially in the realm of extensive data synthesis. This involves a deeper exploration and augmentation of the condensed data to uncover new insights and patterns that might not be readily apparent in the original, more complex form. Digging deeper into the simplified data may help us better grasp the biological or ecological processes reflected in the OTU data. Transitioning from this exploration, the synergy of ML and synthetic data enrichment holds promise for advancing microbiome research. This approach enhances classification accuracy and reveals hidden microbial markers that could prove valuable in clinical practice as a diagnostic and prognostic tool.

## 1. Introduction

Colorectal cancer (CRC) is the third most common type of cancer and the fourth leading cause of cancer-related deaths globally [1]. Studies predict that by 2035, deaths attributed to colon and rectal cancer will increase by 60% in incidence and 71.5% in mortality [2], though these estimates may vary across different countries, depending on socioeconomic status [3]. Most CRC cases consist of adenocarcinomas, which arise from adenomatous polyps (AP) [4], following the adenoma–carcinoma sequence. This sequence suggests that precancerous polyps accumulate mutations over time, increasing the risk of developing invasive adenocarcinoma [5].

Recent research has reinforced the connection between colorectal cancer (CRC) development and the gut microbiome (GM), particularly in the context of dysbiosis—an imbalance in microbial composition and function. The GM includes prokaryotes as well as fungi, archaea, and protists [6]. Dysbiosis, characterised by reduced beneficial microbes, increased harmful ones, and lower microbial diversity, is closely linked to the progression from colorectal adenomas to CRC [7,8,9]. Gut microbes are essential for energy metabolism, gut barrier integrity, and immune modulation [10]. Factors like diet and environment can disrupt the GM, promoting CRC through chronic inflammation, microbial metabolites, and virulence factors [11,12,13]. Notably, the application of artificial intelligence (AI) in microbiota analysis is rapidly evolving, offering promising avenues for both diagnostic and therapeutic strategies in CRC and related diseases [14].

This article builds on our previous study by Russo et al. [4] and enhances the analysis using statistical methods, incorporating synthetic data generation and machine learning (ML) classifiers. Russo et al. [4] conducted an extensive microbial analysis across three sample types—saliva, tissue biopsy, and faeces—to identify bacterial or metabolite biomarkers that could distinguish between CRC, AP, and different TNM stages of CRC. We extended this approach by extracting additional features essential for differentiating between patients diagnosed with AP and CRC from the cited OTU table. These features may be further applied to classification tasks, with potential clinical applications, such as refining screening recommendations based on patterns and markers identified from the synthesised data. Our novel approach integrates data augmentation with deep learning (DL) and ML techniques to extract distinctive features between these datasets.

One of the primary challenges encountered in this study was the significant imbalance in the data. The CRC patient dataset included 114 samples, while the AP dataset contained only 34 samples. Before any classification tasks could be initiated, data augmentation was necessary to address this imbalance. In situations with class imbalance, models tend to favour the majority class, which undermines the classification’s robustness and reliability. In Johnson et al.’s work [15], a strategy was proposed to address this imbalance by generating synthetic samples to balance and expand the OTU table, thus creating equal datasets for both AP and CRC classes.

The data were then classified using a DL algorithm to identify features that differentiate the CRC and AP datasets. These extracted features were subsequently used as input for ML classifiers, such as random forest, XGBoost, and support vector machine, with the primary goal of identifying biomarkers that could be used as screening tools to distinguish AP from CRC.

Following classification, a Shapley Additive Explanation (SHAP) analysis was performed on the most effective classifier to gain insights into the contribution of each feature to the classification decision. This aligns with the principles of explainable artificial intelligence (XAI). Additionally, we compared these features to those altered in our previous study [4] to identify potential overlaps. Confirming the findings of the earlier study could reinforce the significance of these features in CRC diagnosis, providing valuable insights into whether an individual may be at risk of developing the disease. This not only enhances our understanding of the microbial signatures associated with CRC but also highlights the potential for using these features as early detection and risk assessment tools. Implementing screening protocols based on these microbial markers could enable healthcare providers to identify individuals at higher risk of CRC, facilitating timely interventions.

The objective of this research is to develop and validate an ML-based approach for distinguishing between adenomatous polyps (AP) and colorectal cancer (CRC) using microbiome data, with a focus on overcoming data imbalance through synthetic data generation. By enriching a limited and skewed dataset with high-quality synthetic samples and employing advanced classification and feature extraction techniques, this study aims to identify key microbial taxa that can serve as potential biomarkers. This approach enhances the diagnostic potential of microbiome analysis and lays the groundwork for non-invasive, data-driven tools for early CRC detection and risk stratification.

## 2. Materials and Methods

**Figure 1:**
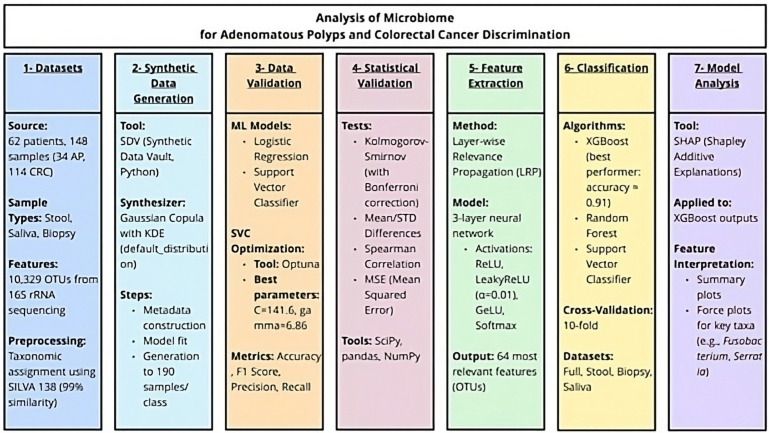
Flowchart of the entire workflow related to the research.

The detailed flowchart that encompasses the entire workflow of the proposed study is shown in Figure 1.

### 2.1. Origin of the Data

**Figure 2:**
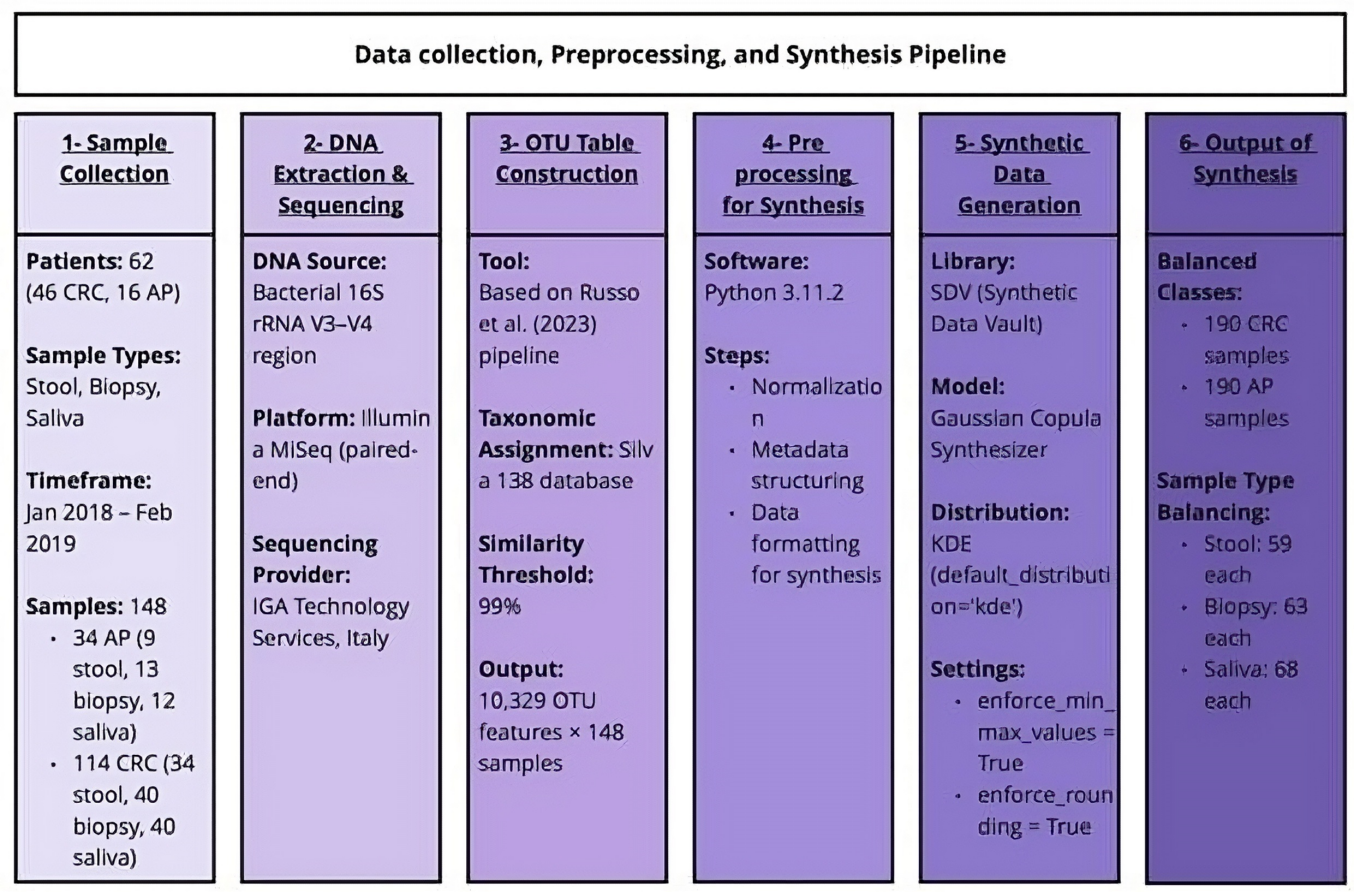
Flowchart summarising the data collection, preprocessing, and synthesis pipeline. The generation of the OTU table is based on the previous work by Russo et al. [4].

The OTU table used in this study originated from an observational research project conducted between January 2018 and February 2019, involving 62 participants. Among them, 46 were diagnosed with CRC and 16 with AP. After sample collection from the patients’ stool, biopsy, and saliva, genomic DNA was extracted and sent to IGA Technology Services in Udine, Italy. Amplicons of the variable V3-V4 region of bacterial 16S rRNA were sequenced in paired-end mode on the Illumina MiSeq platform. Following the bioinformatic analysis performed by Russo et al. [4], an OTU table was generated, and taxonomic assignment for each OTU in the samples was performed using the Silva 138 database, with a 99% similarity threshold. The resulting OTU table consists of 148 samples (34 from AP patients and 114 from CRC patients) and 10,329 features. The 34 samples from AP patients included 9 faecal, 13 biopsy, and 12 saliva samples, while the CRC group included 34 faecal, 40 biopsy, and 40 saliva samples. Figure 2 shows the flowchart summarising the data collection, preprocessing, and synthesis pipeline. All subsequent analyses were carried out using Python v3.11.2.

### 2.2. Data Synthesis

Before describing the actions taken on the dataset, it is important to note that the data were analysed in their raw form, without any prior preprocessing. No normalisation or filtering techniques were applied beforehand. Given the substantial disparity in sample sizes between CRC and AP patients, addressing class imbalance was critical. The Synthetic Data Vault (SDV) Python package was employed to generate synthetic OTU tables. The SDV tool, as described by Hittmeir et al. [16], was originally used for anonymising patients’ microbial data, but in this study, it was focused on balancing and augmenting the dataset. The tool was selected because it had previously been applied to the same type of data—operational taxonomic unit (OTU) counts—in the work of Hittmeir et al. [16], aligning closely with the nature of our dataset.

Initially, data were preprocessed for subsequent DL-based feature extraction and classification tasks to differentiate CRC and AP samples. The SDV’s synthesis procedure involved four steps: organising the data with metadata, initialising the synthesiser (Gaussian Copula) with appropriate parameters (e.g., “enforce_min_max_values” and “enforce_rounding”), constructing the generative model using the command “synthesizer_fit(real_data)” [17], and finally generating synthetic data that mirrored the format of the original dataset.

For this study, the Gaussian Copula synthesiser was configured with the ‘Default_distribution’ parameter set to Gaussian Kernel Density Estimation (KDE) [17], chosen because the data may deviate from a standard normal distribution. After synthesis, both the AP and CRC datasets were expanded to 190 samples each. Specifically, 59 stool samples, 63 biopsy samples, and 68 saliva samples were synthesised. For example, the 12 real saliva samples from AP patients were used to generate an initial round of 12 synthetic samples, followed by additional rounds to reach 68 samples. The same approach was applied to stool and biopsy samples. For CRC samples, 20 real samples from each sample type were used as a basis for generating additional synthetic samples, expanding the dataset to 190 samples per group.

Only 20 CRC samples per type were used for computational efficiency, while all AP samples were included to maximise variability within this smaller group. Importantly, this sampling strategy aimed to preserve representativeness while managing the high dimensionality of the OTU data (10,329 features across 148 samples) and the intensive resource demands of the Gaussian Copula model. To mitigate potential bias, we ensured that the selected CRC samples were randomly chosen and stratified by sample type (stool, biopsy, and saliva), maintaining internal class heterogeneity. Moreover, post-synthesis validation included rigorous statistical and ML-based evaluations (Kolmogorov–Smirnov tests, logistic regression, SVC, etc.—see Section 2.3 and Section 2.4) to confirm the synthetic data’s similarity to the full original dataset. These validations showed no significant statistical deviations, supporting the adequacy of the selected subset.

### 2.3. Data Quality Metrics

After each round of synthesis, data were validated using ML algorithms: logistic regression (LR) and support vector classifier (SVC) with a polynomial kernel, both implemented using the scikit-learn library. These classifiers had been previously evaluated on a dataset formed by biopsy and faecal samples from CRC patients, extrapolated from the total pool of 114 samples.

#### 2.3.1. Support Vector Classifier Optimisation

While LR showed no issues in distinguishing the data, the polynomial kernel of the SVC required fine-tuning to optimise its performance. The Optuna Python package was employed to explore a range of hyperparameters and fine-tune the model. The parameters C and γ, which significantly affect model performance, were optimised based on the dataset [18]. The C parameter range was explored between 0.01 and 1000, while γ was tested between 0.0001 and 100. After running 100 trials, the most suitable parameters were selected to maximise the SVC’s accuracy.

#### 2.3.2. Methods for Data Classification

The classification methodology followed the approach outlined by Chen et al. [19]. Real and synthetic data were merged into a single dataset, with a target variable assigned to differentiate the two. In this study, care was taken to balance real and synthetic datasets to ensure fair classification. The two selected classifiers (LR and SVC) were then fitted in parallel to predict the data’s classes. The goal was to directly assess the models’ ability to distinguish real data from synthetic data in a combined scenario. Before classification, 500 features were selected using the *SelectKBest* function from *scikit-learn*, applying a chi-squared test. Model performance was assessed using F1 score, precision, recall, and accuracy. Synthetic data points that were most difficult to classify were retained to ensure the synthetic data closely resembled real data.

### 2.4. Statistical Analysis

To enhance data validation, the Kolmogorov–Smirnov (KS) test was applied to evaluate whether columns from the real and synthetic datasets followed the same statistical distribution. This was done using the *ks2_samp* function from *SciPy.stats*. To control for multiple comparisons, the Bonferroni correction was applied, ensuring a significance level of 0.05. Additional metrics, such as mean difference, standard deviation (STD) difference, Spearman correlation, and mean squared error (MSE), were calculated. Lower values of mean difference and STD difference indicated smaller discrepancies, while higher MSE values suggested greater dissimilarities between real and synthetic data. The KS test and these metrics were applied by pairing 9, 12, and 13 real stool, saliva, and biopsy samples, respectively, with an equivalent number of synthetic samples from the AP dataset. A similar approach was used for CRC samples. Once validated, synthetic datasets were merged with the real data to equalise the number of AP and CRC samples.

### 2.5. Feature Selection

Layer-wise relevance propagation (LRP) was employed on a deep neural network classification model with a 10-fold cross-validation to identify the key features distinguishing AP from CRC samples. LRP is suitable for neural networks, which may involve various types of input data, such as text or images [20]. A simplified custom version of LRP was deployed using Keras backend functions and NumPy, without relying on external libraries. This allowed having the desired flexibility across multiple activation functions. Specifically, relevance propagation was computed by iterating backwards through the layers and applying a rule akin to the LRP-ε rule.

Feature extraction was performed inside the cross-validation folds on a subset of randomly selected samples (18 records—10% of the whole dataset), balanced across the three specimens (saliva, stool, and biopsy). The remaining 172 samples were reserved for the subsequent training and validation of ML models (Section 2.7). The designed neural network for feature extraction consisted of three layers: two dense layers and a sigmoid activation layer for binary classification. The model’s performance was evaluated using different activation functions (ReLU, LeakyReLU—α = 0.01, Softmax, and GeLU) for the dense layers, while the other hyperparameters were kept constant (Table 1). The best-performing activation function was then selected and applied to the model for feature selection.

The selected features were compared with those identified by Russo et al. [4], and the common features were selected into a new dataset to assess whether the reduced subset retained sufficient information to accurately classify the samples. All further analyses were performed separately on both datasets: the one including all the features extracted with LRP and the one with only the common features.

### 2.6. Beta-Diversity Analysis

A beta-diversity analysis was conducted on both CRC and AP samples, following the method used in Russo et al. [4]. The analysis utilised the scikit-bio library, starting with the square root of the percentage abundance of OTU counts. A Bray–Curtis dissimilarity (BCD) distance matrix was computed, followed by Principal Coordinate Analysis (PCoA) to visualise the sample differences. Samples were colour-coded by their region of origin, with separate visualisations for AP and CRC patients. Both real and synthetic samples were included, and the analysis was performed on datasets containing all 10,329 features, as well as the reduced sets of the whole extracted features and only the common overlapping features.

### 2.7. Classification and SHAP Analysis

The whole dataset was initially split into two subsets with a ratio of 80:20, balanced across the three specimens (saliva, stool, and biopsy). Notably, 80% of the samples were used for training and validation, while the remaining 20% as a holdout dataset for the final test. Three ML algorithms—SVC, XGBoost, and random forest (RF)—were trained and validated with a 10-fold cross-validation on the larger subset. Afterwards, the best performing architecture (i.e., the one with the highest average accuracy) was chosen for the final model. The final model was then trained again from scratch on the same subset, still with a 10-fold cross-validation to reduce overfitting, as well as to minimise bias and variance when estimating the model’s generalisation performance for classification. Finally, the model was validated on the holdout dataset comprising the unseen 20% of the data.

Classification reports and ROC curves were generated to assess model performance.

SHAP values were evaluated for the final model on the whole holdout dataset and for each specimen to explain the positive or negative contributions of each feature to model predictions, providing insights into the most influential features for differentiating CRC and AP.

## 3. Results

### 3.1. Synthetic Data Evaluation

#### 3.1.1. Data Classification

The primary goal of this analysis is to identify distinguishing features between CRC and AP patients. The initial OTU table contained samples from various locales (stool, biopsy, and saliva) and included both AP and CRC patients. To address class imbalance, two balanced datasets were prepared for AP and CRC samples, enhanced with synthetic data. This approach enabled a comprehensive analysis, facilitating feature identification and subsequent classification tasks. The synthetic data were assessed alongside real data using two classifiers. This evaluation involved a combined dataset of real and synthetic samples, simulating a realistic scenario where both data categories coexist.

The support vector classifier was configured with optimised hyperparameters: C = 141.6 and γ = 6.86, based on prior tuning. Detailed classification performance metrics, including accuracy, precision, and recall, are provided in the Supplementary Materials. Data that led to poorer classifier performance was selected for further validation with additional metrics.

#### 3.1.2. Statistical and Quality Assessment

The Kolmogorov–Smirnov test, with Bonferroni correction for multiple comparisons, was applied to the synthetic and real data from stool, biopsy, and saliva datasets of AP and CRC samples. No statistically significant differences were detected in the distributions (*p*-values exceeded alpha = 0.05; details in the Supplementary Materials). Additional metrics, such as mean differences and standard deviation (STD) differences, were also examined.

In CRC datasets, mean differences ranged from 10.65 to 20.26, with STD differences between 20.83 and 33.86. A low positive Spearman correlation (0.01 to 0.05) was observed, and MSEs ranged from 256,682 to 520,054. For AP datasets, mean differences were higher (20.27 to 41.94), with STD differences from 26.82 to 57.86. A negative Spearman correlation (−0.17 to −0.11) was measured for saliva and biopsy datasets, with MSE values ranging from 541,909 to 1,979,617. These values are described qualitatively as “high”, “moderate”, or “low” due to the exploratory nature of the analysis. A table with detailed metrics for real versus synthetic AP and CRC samples is provided in the Supplementary Materials.

### 3.2. Feature Extraction

Sigmoid and hyperbolic tangent activation functions were excluded due to vanishing gradient concerns, as discussed by Nanni et al. [21]. Four activation functions were tested, ReLU, LeakyReLU (alpha = 0.01), Softmax, and GeLU, with LeakyReLU providing the best results. Subsequent cross-validation with the selected activation function yielded an accuracy of 0.79 and a total of 64 extracted features. The 64 features extracted with LRP were those with the top mean LRP score across all features. The selected features were compared with those identified by Russo et al. [4], and 19 common features were found. An additional dataset with only the features in common was created to determine if the reduced subset preserved enough information to appropriately categorise the samples.

### 3.3. Beta-Diversity Insights

**Figure 3:**
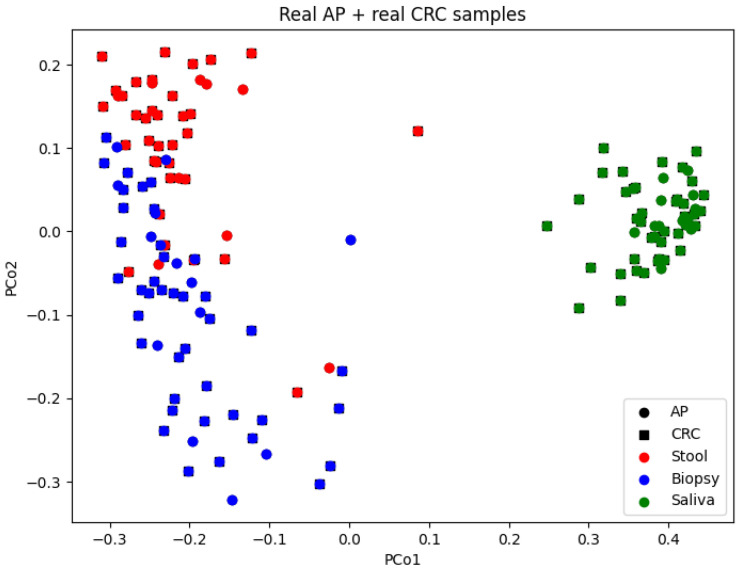
Principal Coordinate Analysis of the original OTU table analysing real AP and CRC samples.

**Figure 4:**
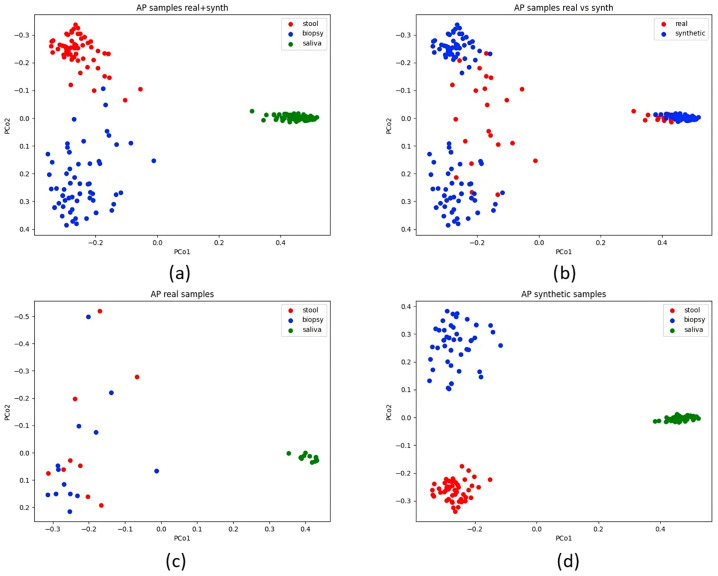
The plot displays the AP dataset encompassing real and synthetic samples. Subfigure (**a**) shows real and synthetic data merged, while in subfigure (**b**), real and synthetic data are differentiated by colour, providing a visual distinction. Subfigures (**c**,**d**) show, respectively, real and synthetic data (the latter is rotated by 180 degrees).

**Figure 5:**
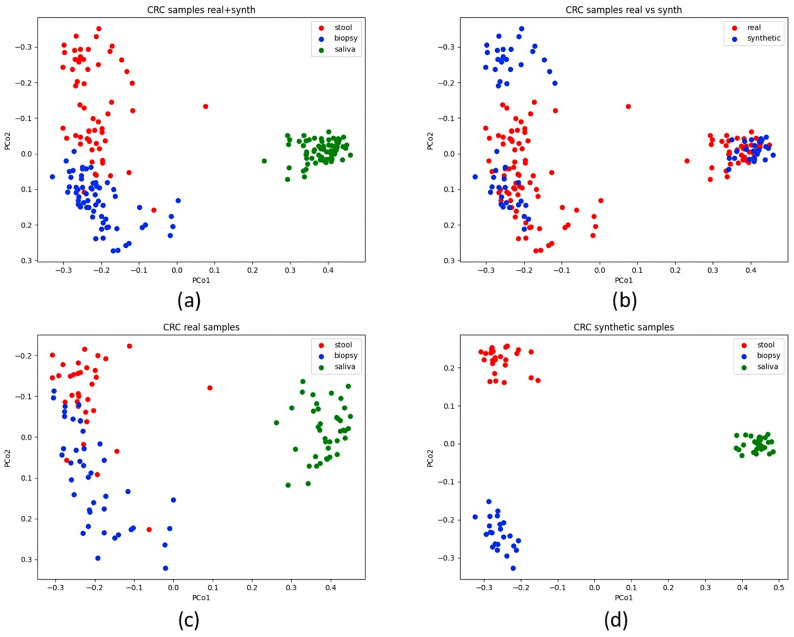
The plot displays the CRC dataset encompassing real and synthetic samples. Subfigure (**a**) shows real and synthetic data merged, while in subfigure (**b**), real and synthetic data are differentiated by colour, providing a visual distinction. Subfigures (**c**,**d**) show, respectively, real and synthetic data.

**Figure 6:**
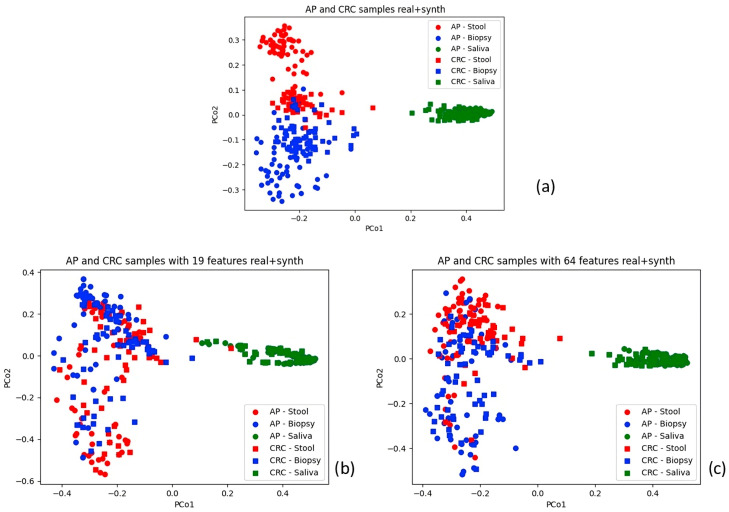
Subfigure (**a**) at the top shows the comprehensive dataset with 10,329 features; subfigures (**b**,**c**) show the 19- and 64-feature datasets, respectively.

Principal Coordinate Analysis (PCoA) was applied to a Bray–Curtis dissimilarity matrix to visualise relationships within the dataset. The comprehensive OTU table for both AP and CRC samples was initially visualised (Figure 3). Additional plots were generated for the AP and CRC datasets enriched with synthetic data, differentiating stool, biopsy, and saliva samples by colour. Real and synthetic data were also visualised separately for AP and CRC patients (Figure 4 and Figure 5). A final visualisation displayed comprehensive datasets, including AP and CRC patients, across the full set of features (10,329) and the reduced sets (19 and 64 features) (Figure 6). The spatial distribution of samples in the principal coordinate space is consistent with the findings of Russo et al. [4], showing distinct microbial compositions between saliva and biopsy/faecal samples.

### 3.4. Classification and SHAP Analysis Outcomes

#### 3.4.1. SHAP Analysis Overview

XGBoost emerged as the best-performing classifier (accuracy of 0.91) when compared to random forest (accuracy of 0.83) and support vector classifier (accuracy of 0.70). This classifier was subsequently used for the classification of datasets separated by sample type. SHAP analysis was performed on the 19- and 64-feature comprehensive datasets to reveal key features influencing the model’s decisions. As previously mentioned, the decision to perform a classification using the 19 taxa shared with Russo et al. [4] was aimed at evaluating whether this subset alone could effectively separate the samples, comparable to the performance achieved using the full set of extracted features. SHAP summary plots illustrate how each taxon affects predictions, ordering features by relevance. The x-axis represents feature impact on model predictions, while the y-axis shows feature values. A force plot demonstrates how individual features influence predictions, with orange regions indicating positive contributions and purple regions indicating negative effects. In the next section, the top two features from the summary plots for the 19- and 64-feature datasets are described.

#### 3.4.2. Classification and SHAP Analysis of the 19-Feature Dataset

**Figure 7:**
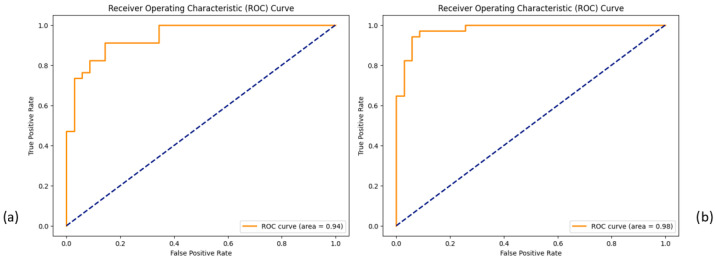
ROC curve for classification using the 19-feature dataset in subfigure (**a**) and the 64-feature dataset in subfigure (**b**).

**Figure 8:**
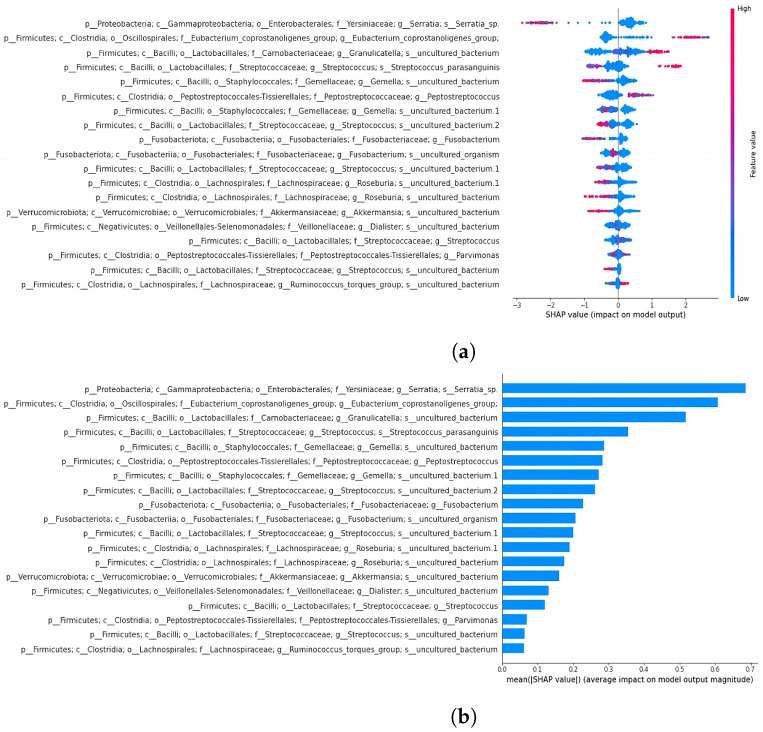
SHAP summary bar plot of 19-feature dataset classification is shown in subfigure (**a**), followed by the second summary beeswarm plot in subfigure (**b**). For the second summary plot, the x-axis represents the SHAP value (impact on model predictions), and the y-axis describes feature values. Each sample is depicted as a red or blue dot.

The XGBoost classifier achieved 0.87 accuracy and an ROC AUC of 0.94 on the 19-feature dataset (Figure 7). The SHAP summary plot (Figure 8) identified key features, including taxa from the family Yersiniaceae (genus *Serratia*), genus *Eubacterium coprostanoligenes* group, family Carnobacteriaceae (genus *Granulicatella*), and family Streptococcaceae (genus *Streptococcus*), among others. The second SHAP summary plot (Figure 8) highlighted *Serratia* and *Eubacterium coprostanoligenes*, both of which influenced model predictions significantly.

#### 3.4.3. Classification and SHAP Analysis of the 64-Feature Dataset

**Figure 9:**
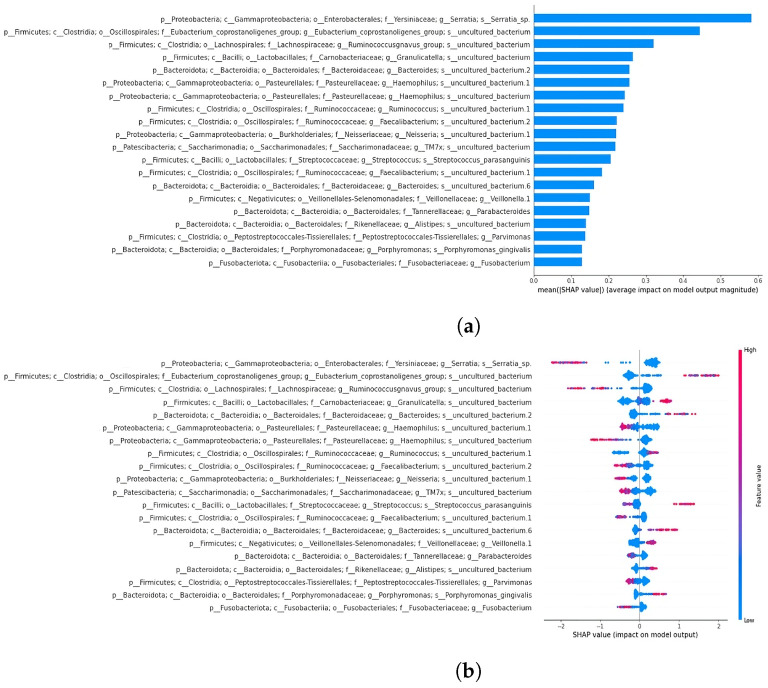
SHAP summary bar plot of the 64-feature dataset classification is shown in subfigure (**a**), followed by the summary beeswarm plot in subfigure (**b**). For the second summary plot, the x-axis represents the SHAP value (impact on model predictions), and the y-axis describes feature values. Each sample is depicted as a red or blue dot.

Expanding the feature set to 64 increased the model accuracy to 0.91 and the ROC AUC to 0.98 (Figure 7). The SHAP summary plot (Figure 9) indicated that taxa from the Yersiniaceae family (genus *Serratia*), the *Eubacterium coprostanoligenes* group, and the *Ruminococcus gnavus* group were among the top six contributors. Additional important features included Bacteroides and Neisseria. Notably, Parvimonas and Fusobacterium appeared as significant features in both LRP and SHAP analyses, reinforcing their roles in model behaviour.

#### 3.4.4. Force Plot of the 64-Feature Dataset

**Figure 10:**
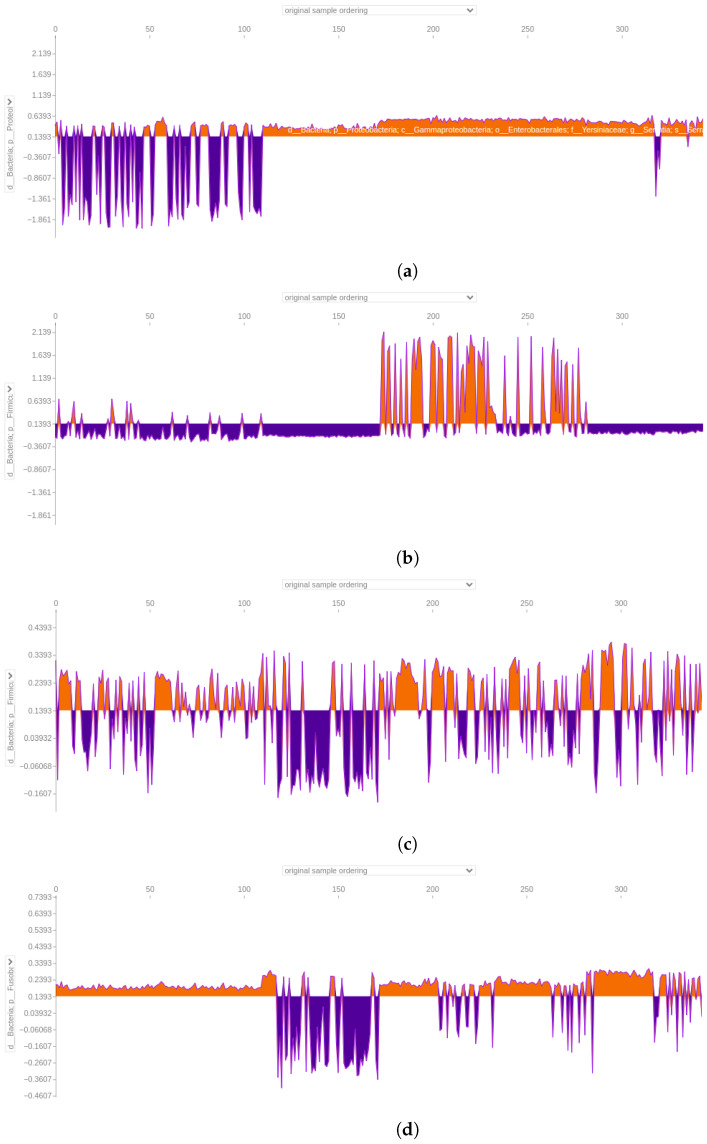
Force plot emphasising the impact of a member of the *Serratia* genus (**a**), the *Eubacterium coprostanoligenes* genus (**b**), the *Parvimonas* (**c**), and the *Fusobacterium* genus (**d**) in predicting adenomatous polyps (AP) and colorectal cancer (CRC) in the 64-feature dataset. Orange positive or purple negative values indicate a positive or negative effect on predicting the sample class. The first 172 samples represent AP samples, followed by 172 CRC samples.

The force plot analysis focused on the top four taxa identified by SHAP in Figure 10. Taxa like *Serratia* and *Eubacterium coprostanoligenes* exhibited positive predictive power for CRC samples and negative effects for AP samples. The same was true for *Ruminococcus gnavus* and *Granulicatella*. Both *Parvimonas* and *Fusobacterium* displayed mixed effects, reinforcing their complex roles in distinguishing between AP and CRC samples.

#### 3.4.5. Classification and SHAP for Individual Datasets

SHAP analysis was also performed on the classification of stool, biopsy, and saliva datasets with the 64 features separately. In the next section, the features are arranged in order of their significance as per the initial summary plot, and each feature is presented alongside its effect on the corresponding force plot. Concerning the depiction of the force plot, reference is directed toward the initial two taxa as arranged in the summary plot, while the effect of the following four taxa will only be described, and the plot is added as Supplementary Materials. We chose to describe the first six force plots for the stool and biopsy dataset. For the second summary plot, figures are presented and the first two taxa behaviours are described.

#### 3.4.6. Stool Dataset

**Figure 11:**
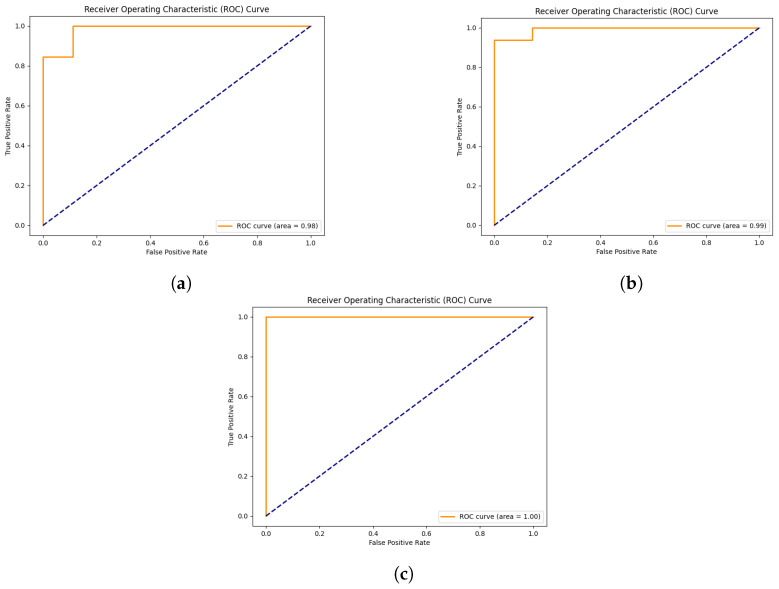
ROC curve for classification using the 64-feature dataset. Subfigure (**a**) shows the ROC curve for the stool dataset, while subfigure (**b**) shows the ROC for the biopsy dataset. In subfigure (**c**), the ROC curve for the saliva sample is shown.

**Figure 12:**
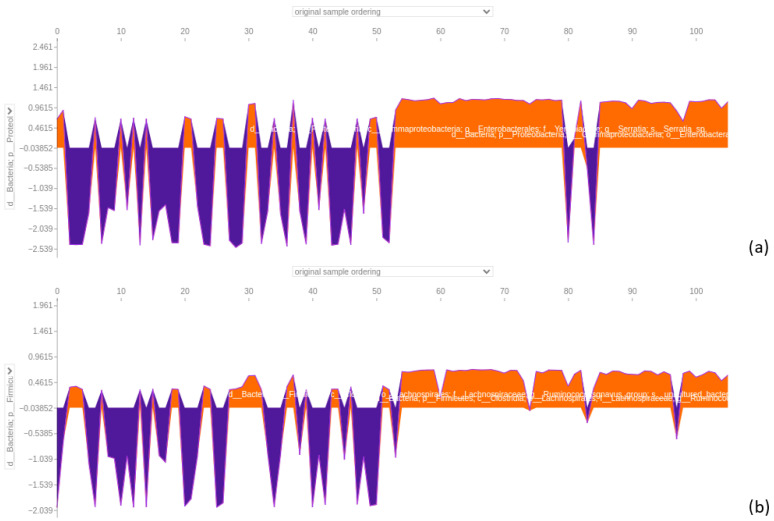
Force plot visualising how a member of the *Serratia* genus in (**a**) and a member of *Ruminococcus gnavus* group in (**b**) influence predicting adenomatous polyps (AP) and colorectal cancer (CRC) in the 64-feature stool dataset. Orange positive or purple negative values indicate a positive or negative effect on predicting the samples’ class. The first 53 samples represent AP samples, followed by 53 CRC samples.

**Figure 13:**
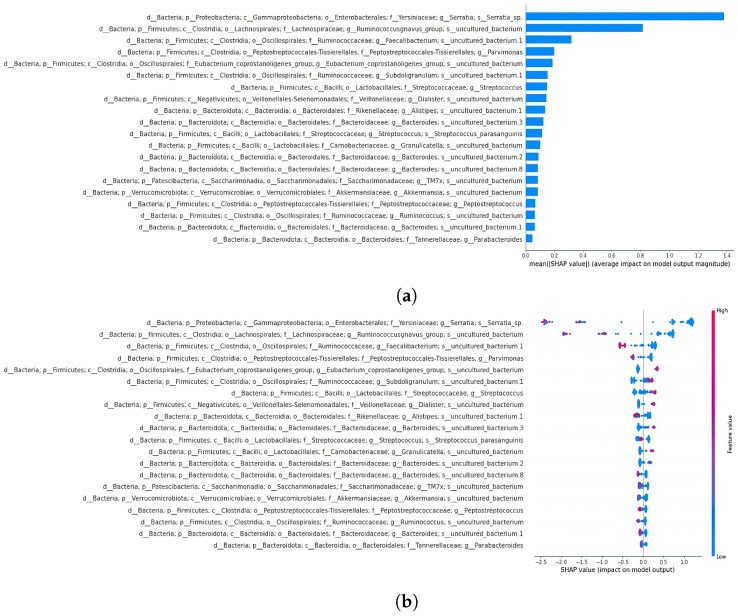
Subfigure (**a**) shows the summary bar plot of the stool dataset, while subfigure (**b**) depicts the summary beeswarm plot.

In the 64-feature stool dataset, classified with an accuracy of 0.86 and an area under the curve of 0.98 (Figure 11), a member of the *Serratia* genus consistently emerged as the primary discriminative feature, strongly supporting CRC predictions. On the contrary, when dealing with AP samples, it negatively affected the predictions, pointing toward the other class (Figure 12). In the second position, the *Ruminococcus gnavus* group showed a similar trend (Figure 12). Two members of Ruminococcaceae appeared in a high position, with one of the *Faecalibacterium* genus being in third place, overall pushing the models toward CRC predictions (see Supplementary Materials). The other member, namely *Subduligranulum* genus, occupied the sixth position, presenting a mix of positive and negative effects, for both the classes of samples (see Supplementary Materials). The *Parvimonas* genus was listed in the fourth position and had mixed positive and negative effects in predicting CRC and AP based on the sample (see Supplementary Materials). Likewise, among features included as most important in this SHAP analysis, the *Eubacterium coprostanoligenes* genus was figured in fifth position, exhibiting distinct positive effects on CRC predictions and negative effects on AP predictions (see Supplementary Materials). About the second summary plot, the focus will be on describing only the initial two taxa from the first summary plot: the *Serratia* genus and the *Ruminococcus gnavus* group. The *Serratia* genus positively influenced model decisions when assuming low feature value, and it had a negative impact on model decisions with a mix of low feature and high feature values. On the other hand, the second taxon, the *Ruminococcus gnavus* group, followed a similar pattern to *Serratia*, albeit with a reduced positive impact and lower negative impact (Figure 13).

#### 3.4.7. Biopsy Dataset

**Figure 14:**
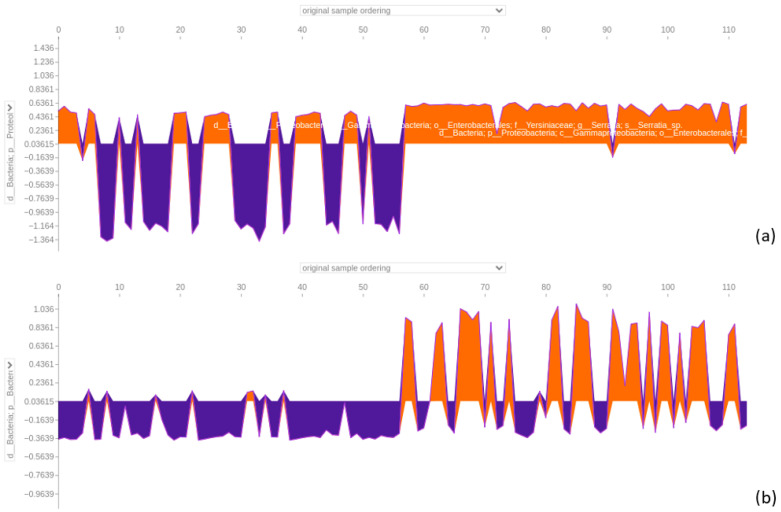
Force plot visualising how a member of the *Serratia* genus in (**a**) and a member of the *Bacteroides* genus in (**b**) influence predicting adenomatous polyps (AP) and colorectal cancer (CRC) in the 64-feature biopsy dataset. Orange positive or purple negative values indicate a positive or negative effect on predicting the samples’ class. The first 57 samples represent AP samples, followed by 57 CRC samples.

**Figure 15:**
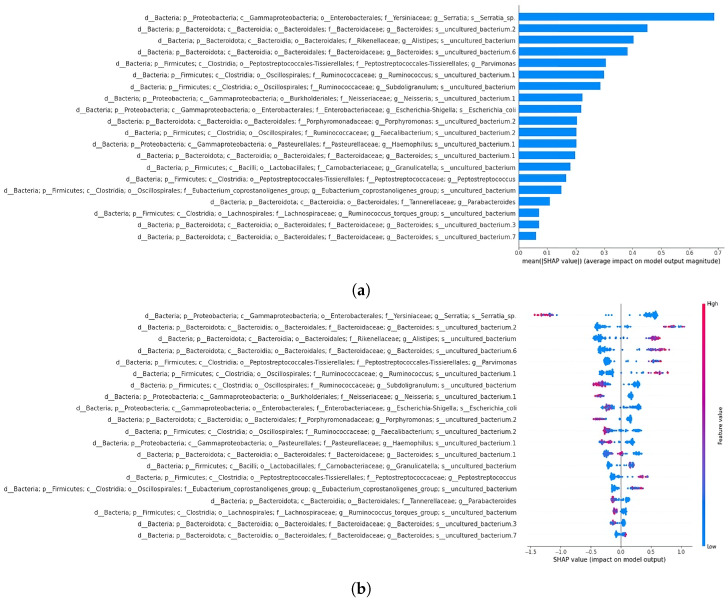
Subfigure (**a**) shows the summary bar plot of the biopsy dataset, while subfigure (**b**) depicts the summary beeswarm plot.

The 64-feature biopsy dataset achieved a classification accuracy of 0.96 with an area under the ROC curve of 0.99 (Figure 11). Notably, the *Serratia* genus remained a primary key feature, favouring CRC predictions and adversely affecting AP predictions, as observed in Figure 14. Following closely is a member of the *Bacteroides* genus, showing a similar trend for AP samples with a more discontinuous positive effect on predicting CRC samples (Figure 14). In the third position, a member of the *Alistipes* genus exhibited a mainly positive impact in predicting CRC samples and a negative impact on AP samples, as shown in the Supplementary Materials. Another Bacteroides member followed, guiding the model toward predicting CRC in both AP and CRC samples. In the fifth position, a member of the *Parvimonas* genus adversely affected AP samples, pointing toward CRC prediction while positively influencing CRC samples (see Supplementary Materials). The sixth and seventh positions feature two members of the Ruminococcaceae family, namely the *Ruminococcus* and *Subdoligranum* genera, impacting both AP and CRC predictions positively or negatively, depending on the sample (see Supplementary Materials). In the context of the second summary plot focusing on *Serratia* and a *Bacteroides* genus member, it was evident that *Serratia* directly modelled positive prediction when assuming low feature values, and on the other hand, it negatively influenced the model when assuming high feature values. The *Bacteroides* genus members behaved in opposite directions, positively influencing the model when assuming high feature values and slightly negatively when assuming low feature values (Figure 15).

#### 3.4.8. Saliva Dataset

**Figure 16:**
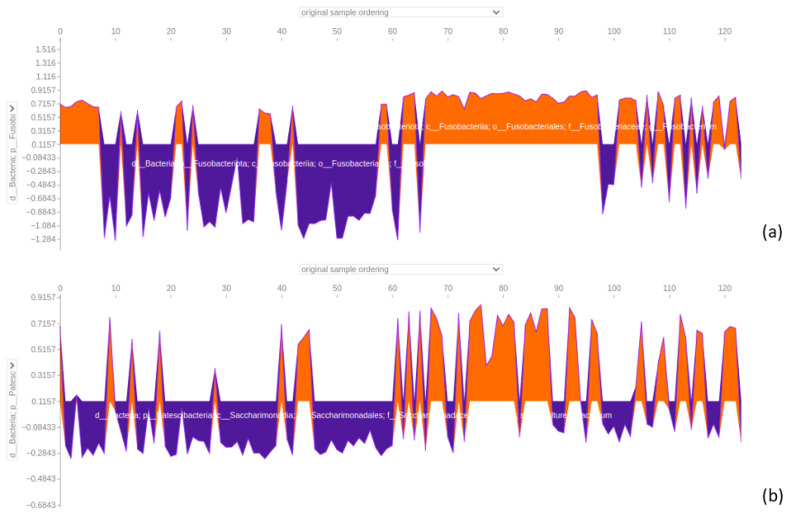
Force plot visualising how a member of the *Fusobacterium* genus in (**a**) and a member of the Saccharimonadaceae family in (**b**) influence predicting adenomatous polyps (AP) and colorectal cancer (CRC) in the 64-feature saliva dataset. Orange positive or purple negative values indicate a positive or negative effect on predicting samples’ class. The first 62 samples represent AP samples, followed by 62 CRC samples.

**Figure 17:**
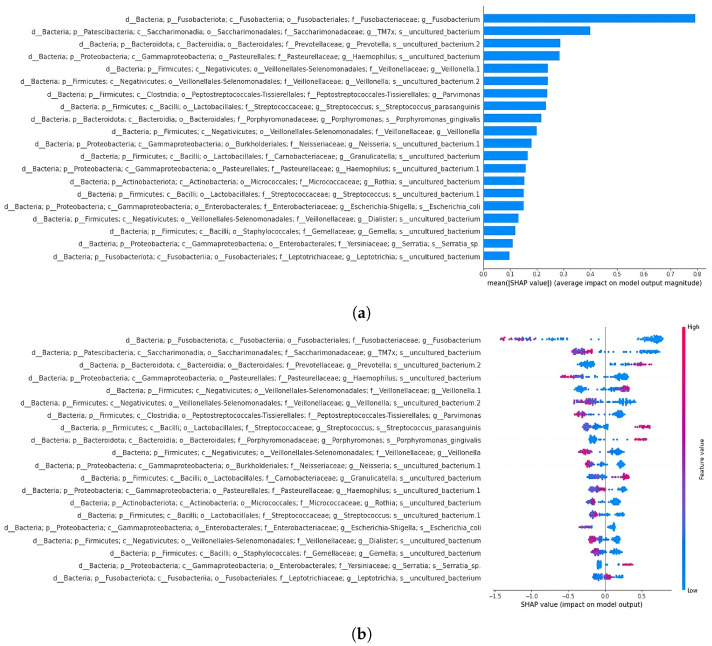
Subfigure (**a**) shows the summary bar plot of the saliva dataset, while subfigure (**b**) depicts the summary beeswarm plot.

When considering saliva samples, the dataset was classified with an accuracy of 1 and an area under the ROC curve of 1 (Figure 11). The SHAP analysis revealed that the top three positions were occupied by distinct microbial taxa. Firstly, a member of the genus Fusobacterium held the highest significance, significantly impacting the prediction of CRC samples. On the contrary, its influence was markedly opposite when it came to almost all AP samples (Figure 16). Additionally, focusing on Figure 16, a representative of the family Saccharimonadaceae took the second position, exhibiting a mixed overall positive effect in predicting CRC samples and a negative effect for AP samples. Lastly, a member of the genus *Prevotella*, positioned at the third spot, mirrored a similar dualistic trend, exerting either a positive or negative influence on the predictions based on the sample type (see Supplementary Materials). Among other features distinguishing saliva samples, it was possible to find the genus *Haemophilus*, *Rothia*, *Veillonella*, *Neisseria*, and *Porhyrimonas gingivalis*, which are taxa commonly found in the human oral cavity [22]. Considering the second summary plot, the *Fusobacterium* genus exerted a positive impact with high SHAP values when assuming low feature values, and it had a positive effect for high feature values. Meanwhile, a member of the Saccharimonadaceae family had a positive impact on the model with low feature values, and it had a slightly positive effect on model decisions with a mix of low and high feature values (Figure 17).

## 4. Discussion

Accumulating evidence suggests that the gut microbiome (GM) plays a central role in the development of colorectal malignancies and the progression of adenomatous polyps (AP) into colorectal cancer (CRC) [9,12]. Understanding disparities in microbial compositions between these conditions can offer valuable insights into the mechanisms driving colorectal malignancy, potentially leading to targeted interventions and a deeper understanding of the complex relationship between gut microbiota and colorectal health.

In this study, we applied synthetic data generation to balance and expand an operational taxonomic unit (OTU) table containing samples from the saliva, stool, and biopsy of patients with CRC and AP. Feature extraction was used to create a condensed representation, highlighting the most relevant features for distinguishing between the two diseases. Balancing and reducing the dataset enabled ML classification tasks to be performed with greater equity between CRC and AP classes, leading to more reliable and unbiased results.

Integrating synthetic data into real-world contexts presents challenges, particularly regarding the potential impact of poor synthetic data quality. If the synthetic data are of low quality, it may introduce uncertainties and alter the outcomes of the analysis. It is important to distinguish between results that reflect real-world scenarios and those influenced by the addition of synthetic data [23].

### 4.1. Evaluation of Classifier Performance and Additional Metrics for Synthetic Data

As outlined earlier, synthetic data were evaluated using SVC and LG. Only the data with the poorest classification performance were selected for further validation. The results of the KS test consistently yielded *p*-values exceeding the alpha threshold of 0.05, indicating that the synthetic and real datasets (stool, biopsy, and saliva) showed statistical similarities.

Regarding additional metrics, both real and synthetic datasets of AP and CRC samples showed weak monotonic relationships and high mean squared errors (MSEs). More pronounced discrepancies were observed in the AP datasets, with the saliva and biopsy samples showing negative Spearman correlations and higher MSEs compared to CRC. However, the low mean and standard deviation differences for both AP and CRC samples suggest greater similarity, particularly in the CRC samples.

### 4.2. Feature Extraction and Identification of Relevant Bacterial Taxa

Feature extraction was performed using a deep learning classification model with the LeakyReLU activation function, which produced the best classification report, with an accuracy of 0.79. LRP was applied to identify 64 features (corresponding to unique bacterial taxa) from the total of 10,329 features.

Among the extracted features, 19 belong to bacterial families or genera that were previously reported in our earlier study [4] to exhibit abundance changes when comparing AP and CRC. These taxa include two members of the Gemellaceae; five members of Streptococcaceae; one member of Yersiniaceae (genus *Serratia*); and additional members of Peptostreptococcaceae, *Dialister*, *Roseburia*, Carnobacteriaceae, *Akkermansia*, *Parvimonas*, *Ruminococcus torques*, *Eubacterium coprostanoligenes*, and *Fusobacterium*.

### 4.3. Discrepancies in PCoA Patterns: Real vs. Synthetic AP Samples

When examining the PCoA plot of AP samples (both real and synthetic), it is evident that the synthesiser struggled to replicate the similarity between real biopsy and faecal samples. This was true for both AP and CRC datasets with all starting features. The disparity became more pronounced when plotting only synthetic data, resulting in a clear separation between CRC and AP samples (Figure 4 and Figure 5). Interestingly, in the comprehensive dataset with 64 and 19 features, faecal and biopsy samples appeared more mixed, suggesting a similar microbial composition. However, the 19-feature dataset showed some interference between biopsy and stool samples with saliva samples (Figure 6). AP saliva samples also exhibited significantly less variability than CRC saliva samples, potentially contributing to the distinctiveness of saliva samples in the classification analysis.

### 4.4. XGBoost Classifier Dominance in Sample Segregation and Feature Distinction

The XGBoost classifier consistently outperformed RF and SVC in distinguishing between AP and CRC across the comprehensive datasets with 19 and 64 features (see Supplementary Materials). With accuracies of 0.87 and 0.91, respectively, XGBoost emerged as the top-performing classifier and was thus used to segregate the dataset based on sample type. For the 64-feature dataset, XGBoost achieved accuracies of 0.86, 0.96, and 1.00 for stool, biopsy, and saliva samples, respectively (Figure 11).

These accuracy results highlight the classifier’s ability to distinguish saliva and biopsy samples between AP and CRC, while faecal samples exhibited comparatively lower discernibility. The features derived from LRP and the shared features show promise as valuable bacterial taxa for effectively distinguishing AP from CRC and could potentially serve as screening tools for CRC diagnosis in clinical settings.

### 4.5. SHAP Analysis Reveals Discrepancies in Feature Importance: A Closer Look at XGBoost Classification of the 64-Feature Dataset

In the SHAP analysis of the 64-feature dataset classified by XGBoost, fewer common features were identified compared to the LRP analysis in our previous study [4]. Notable taxa include *Serratia*, *Eubacterium coprostanoligenes*, Carnobacteriaceae, Peptostreptococcales-Tissierellales (genus *Parvimonas* in 18th position), and *Fusobacterium* (in 20th position). The SHAP plot also highlighted taxa from the oral cavity, including *Haemophilus*, *Neisseria*, *Porphyromonas gingivalis*, and *Fusobacterium* (Figure 9). Since previous studies found no significant differences in microbial abundances between AP and CRC saliva samples, the differences observed in this study could potentially be attributed to the use of synthetic data. The reduced variability in AP saliva samples compared to CRC samples (as seen in the PCoA plot) may have contributed to the classification differences.

### 4.6. Significance of Fusobacterium and Parvimonas Genus

The Fusobacterium genus appears as a significant feature in both DL and ML classifications of the 64- and 19-feature datasets, suggesting it may serve as a key distinguishing factor. As noted in the study of Russo et al. [4], *Fusobacterium* is associated with CRC and tends to increase during cancer development. Similar observations can be made for the Parvimonas genus, which appears as a distinguishing feature in both the stool and biopsy datasets (Figure 10).

The *Fusobacterium* force plot reveals that it is a negative predictor for saliva AP samples, while *Parvimonas* displays a more inconsistent pattern, alternating between positive and negative effects depending on the sample type.

### 4.7. Comparisons of Taxa with Previous Findings

A comparison with taxa distinguishing CRC and AP for stool, biopsy, and saliva samples separately was conducted to compare the results of our previous study [4].

#### 4.7.1. Similarities in Faecal Microbiota

Regarding faecal samples, previous studies found significant differences in several genera, including *Akkermansia*, *Anaerostipes*, *Bifidobacterium*, *Dialister*, and *Eubacterium coprostanoligenes*, between CRC and AP patients. Our findings confirmed the importance of *Eubacterium coprostanoligenes* as a distinguishing feature, as evidenced by its fifth position in the SHAP plot (Figure 13). When considering *Akkermansia*, it drives the model toward AP or CRC predictions inconsistently, while the effect of *Dialister* seems to pinpoint the model in predicting CRC (see Supplementary Materials).

#### 4.7.2. Similarities in Biopsy Microbiota

Previous studies in biopsy samples reported increases in Peptostreptococcales-Tissierellales, Carnobacteriaceae, Gemellaceae, and Streptococcaceae in CRC. Our SHAP analysis highlighted two members of Peptostreptococcales-Tissierellales, a member of Carnobacteriaceae (*Granulicatella* genus), and *Serratia* (Yersiniaceae family) as key discriminating features between CRC and AP (Figure 14). Serratia emerged as the primary discriminating feature in both the stool and biopsy datasets, as well as in the SHAP analysis of the 64-feature comprehensive dataset. Overall, this taxon tends to act as a negative predictor for AP samples and a positive predictor for CRC samples. Moreover, the member of the *Parvimonas* genus, in the biopsy dataset, pushes the model to predict the CRC class, for both AP and CRC samples, while the *Granulicatella* genus has a discontinuous effect on model predictions, with a mix of positive or negative effects on AP and CRC biopsy samples (see Supplementary Materials).

#### 4.7.3. Insights from Key Genera in Stool and Biopsy Samples

Alistipes emerged as a determinant feature in both stool and biopsy datasets (Figure 13 and Figure 15). In addition, several members of the Ruminococcaceae family, particularly within the *Ruminococcus*, *Faecalibacterium*, and *Subdoligranulum* genera, appear among the most determining features of both faecal and biopsy samples. This observation aligns with findings from Liu et al. [24], who reported that gut microbiota (GM) abundance and diversity may increase gradually with CRC occurrence and progression. Specifically, their analysis of faecal samples indicated that elevated abundances of *Alistipes* and *Ruminococcus* may contribute to CRC progression, suggesting that these taxa may serve as discriminative features between advanced polyp (AP) and CRC conditions. Alistipes has been associated with pro-inflammatory activity and the promotion of a tumorigenic microenvironment, possibly through the production of sulfides and other harmful metabolites [25,26]. Members of *Ruminococcus*, particularly *Ruminococcus gnavus*, have been linked to impaired mucosal integrity and immune modulation, which may facilitate colorectal tumorigenesis [27]. Additionally, a member of the genus *Parvimonas*, which ranked fourth and fifth in importance in the stool and biopsy SHAP summary plots, respectively, has previously been proposed as a non-invasive faecal biomarker for CRC detection [28]. *Parvimonas micra*, in particular, has been found enriched in CRC patients and may contribute to disease progression by enhancing inflammatory responses and modulating immune signalling pathways [29]. Notably, some of these microbial taxa, including *Alistipes* and *Parvimonas micra*, have also been detected at elevated levels in individuals with adenomatous polyps, suggesting that their presence may mark an early shift in microbial composition associated with the adenoma–carcinoma sequence [30]. Their enrichment in AP samples supports the hypothesis that microbial dysbiosis begins before malignant transformation and could play a role in initiating or sustaining early neoplastic changes.

#### 4.7.4. Similarities in Saliva Microbiota

Regarding saliva samples, as mentioned earlier, Russo et al. [4] did not find statistical differences between taxa in AP and CRC samples. However, in this study, Xgboost classification achieved maximum accuracy, indicating significant distinctions between the two sample classes. These findings hold significant implications for clinical applications, suggesting that a straightforward saliva test could effectively distinguish between CRC and AP with the aim of our extracted feature and our trained Xgboost classifier. However, it is worth mentioning that the previously cited reduced variability in saliva samples of AP poses challenges, introducing a potential constraint on the overall reliability of the obtained results. There is certainly a need for new sample collection to enhance the comprehensiveness and accuracy of the analysis, especially when considering the classification of the saliva dataset.

#### 4.7.5. Insights from Key Genera in Saliva Samples

The primary discriminator in our data is *Fusobacterium*, which exerts a negative influence on AP predictions, and a positive effect on CRC predictions (Figure 16). This seems to be in line with the findings of Zhang et al. [31], where it was documented that the relative level of *Fusobacterium nucleatum* DNA species increased in the saliva of the CRC group compared to normal colonoscopy, hyperplastic polyp, and adenoma groups. However, caution is needed in interpreting our findings, as previously reported, no statistical differences were found in saliva samples between AP and CRC patients, even if they were dealing with limitations caused by data disparity. Further research and validation are crucial to reconcile these disparities and gain a comprehensive understanding of *Fusobacterium*’s role in distinguishing between AP and CRC in saliva samples. Importantly, Fusobacterium nucleatum has been implicated in the pathogenesis of both AP and CRC. It has been shown to promote tumorigenesis by adhering to and invading epithelial cells, activating β-catenin signalling, and modulating host immunity through the suppression of T-cell-mediated responses [32,33]. Furthermore, its presence has been associated with increased expression of pro-inflammatory cytokines and enrichment in precancerous lesions, such as adenomas, suggesting a potential role in the early stages of the adenoma–carcinoma sequence [34,35]. These findings support the hypothesis that *F. nucleatum* may contribute to the transition from benign polyps to malignancy by creating a pro-inflammatory, immunosuppressive microenvironment favourable to tumour development.

The various force plots regarding the effect of each common feature, along with the article of Russo et al. [4] in stool, biopsy, and saliva datasets, are available in the Supplementary Materials.

## 5. Limitations

One key limitation of this research lies in the reliance on synthetic data to balance the dataset, which, while effective in enhancing classifier performance, may introduce artefacts that do not fully reflect the complexity of real biological variation. Although extensive validation was performed using statistical tests and classification metrics, synthetic samples might fail to capture the full range of diversity, especially in the underrepresented adenomatous polyp (AP) group. Moreover, the KS test has been used with a relatively small subsample size (*n* = 9–13). The statistical power of the KS test is sensitive to sample size, and small samples can substantially reduce its ability to detect true differences between distributions. As a result, there is an increased risk of Type II errors (i.e., failing to detect a difference when one exists), potentially leading to underestimation of variability or similarity across conditions. Another limitation is the treatment of multiple samples from the same patient (i.e., stool, biopsy, and saliva) as independent data points. This approach neglects intra-patient correlations and may inflate the statistical power of the models while introducing bias, as samples from the same individual are not truly independent. Furthermore, the study lacks a healthy control group, restricting the ability to differentiate disease-specific microbial shifts from general features of gut dysbiosis. Without healthy baseline comparisons, microbial markers identified as discriminative between AP and colorectal cancer (CRC) may simply reflect broader alterations associated with illness rather than specific stages of disease progression. Lastly, the relatively small and imbalanced original dataset limits the generalisability of the findings. The small number of available samples hampered the implementation of a larger independent test set, which could result in an overestimation of the models’ performance and the possibility of data leakage. These limitations highlight the need for expanded datasets and multi-centre studies to confirm the clinical utility of the proposed approach. Future work should include a larger, more diverse cohort, incorporate healthy controls and additional or complementary distributional similarity metrics, and account for intra-patient variability to strengthen the clinical relevance and robustness of the results.

## 6. Conclusions

This study integrates synthetic data generation and machine learning to distinguish between AP and CRC in human samples. By utilising support vector classification and logistic regression as primary methods for generating realistic synthetic data, we developed a balanced dataset that enhanced the analysis. Deep learning techniques, specifically layer-wise relevance propagation (LRP), proved effective in identifying 64 distinctive bacterial taxa, of which 19 align with findings from relevant studies, underscoring their potential significance in distinguishing between AP and CRC. These 64 features also show promise for CRC screenings, suggesting their utility in early detection and diagnosis efforts.

Key findings emphasise the importance of the *Fusobacterium* genus, which was consistently highlighted in both LRP and SHAP analyses due to its known association with CRC. Through SHAP analysis of the XGBoost classifier, additional discriminating features, such as *Parvimonas*, *Alistipes*, and members of the Ruminococcaceae family, emerged, providing valuable insights into microbial compositions linked to CRC.

Despite the computational challenges posed by synthesising data using the Synthetic Data Vault, the reduced feature set post-extraction allows for further exploration of synthesis methods. However, caution is necessary when working with synthetic data due to potential uncertainties, underscoring the importance of comparing classifier performance on real and synthetic datasets to ensure reliability.

In conclusion, our approach advances CRC diagnosis and screening through the integration of synthetic data and machine learning, highlighting the power of these tools in enhancing our understanding of complex microbiota ecosystems and their role in human health disorders. This study opens new avenues for utilising synthetic data and microbial markers in early detection and personalised healthcare.

## Figures and Tables

**Table 1:** List of the tested hyperparameters for training the custom LRP model for feature selection.

Hyperparameter	Tested Values
Model Architecture	Dense (64) → ReLU/LeakyReLU (α = 0.01)/Softmax/GeLU Dense (32) → ReLU/LeakyReLU (α = 0.01)/Softmax/GeLU Dense(1,Sigmoid)
Optimizer	Adam
Loss Score	Binary Crossentropy
Batch Size	32
Epochs	45

## Data Availability

The datasets for this study are accessible at https://github.com/alextopology/microbiome-analysis/tree/main (accessed on 26 June 2025).

## Supplementary Materials

The following supporting information can be downloaded at: https://www.mdpi.com/article/10.3390/bioengineering12070713/s1.

## Abbreviations

The following abbreviations are used in this manuscript:

AP	Adenomatous Polyps
AUC	Area Under the Curve
BCD	Bray–Curtis Dissimilarity
CRC	Colorectal Cancer
DL	Deep Learning
GeLU	Gaussian Error Linear Unit
KDE	Kernel Density Estimation
KS	Kolmogorov–Smirnov (test)
LeakyReLU	Leaky Rectified Linear Unit
LR	Logistic Regression
LRP	Layer-Wise Relevance Propagation
ML	Machine Learning
MSE	Mean Squared Error
OTU	Operational Taxonomic Unit
PCoA	Principal Coordinates Analysis
ReLU	Rectified Linear Unit
RF	Random Forest
ROC	Receiver Operating Characteristic
SDV	Synthetic Data Vault
SHAP	SHapley Additive exPlanations
STD	Standard Deviation
SVC	Support Vector Classifier
XAI	eXplainable Artificial Intelligence
