# Supplementary material for: Analysis of Microbiome for AP and CRC Discrimination
Source: Bioengineering (Basel). 2025 Jun 29;12(7):713. doi: 10.3390/bioengineering12070713 (PMC12292819; doi:10.3390/bioengineering12070713)
Supplement: Supplementary file 1 [file bioengineering-12-00713-s001.zip › bioengineering-3634846-supplementary.pdf]

Classification reports of datasets containing real and synthetic data

a)

	precision	recall	f1-score	support
0	0.00	0.00	0.00	3
1	0.25	0.33	0.29	3
accuracy			0.17	6
macro avg	0.12	0.17	0.14	6
weighted avg	0.12	0.17	0.14	6

	precision	recall	f1-score	support
0	0.33	0.33	0.33	3
1	0.33	0.33	0.33	3
accuracy			0.33	6
macro avg	0.33	0.33	0.33	6
weighted avg	0.33	0.33	0.33	6

b)

	precision	recall	f1-score	support
0	0.20	0.33	0.25	3
1	0.33	0.20	0.25	5
accuracy			0.25	8
macro avg	0.27	0.27	0.25	8
weighted avg	0.28	0.25	0.25	8

	precision	recall	f1-score	support
0	0.33	0.67	0.44	3
1	0.50	0.20	0.29	5
accuracy			0.38	8
macro avg	0.42	0.43	0.37	8
weighted avg	0.44	0.38	0.35	8

Classification report of real and synthetic AP samples of stool, subfigure a) and biopsy, subfigure b) samples of the classifiers Logistic regression (on top) and support vector classifier (On bottom)

	precision	recall	f1-score	support
0	0.25	0.25	0.25	4
1	0.00	0.00	0.00	3
accuracy			0.14	7
macro avg	0.12	0.12	0.12	7
weighted avg	0.14	0.14	0.14	7

	precision	recall	f1-score	support
0	0.33	0.25	0.29	4
1	0.25	0.33	0.29	3
accuracy			0.29	7
macro avg	0.29	0.29	0.29	7
weighted avg	0.30	0.29	0.29	7

Classification report of real and synthetic AP samples on saliva samples of Logistic Regression (On top) and Support vector classifier (on bottom).

a)

	precision	recall	f1-score	support
0	0.33	0.20	0.25	5
1	0.20	0.33	0.25	3
accuracy			0.25	8
macro avg	0.27	0.27	0.25	8
weighted avg	0.28	0.25	0.25	8

	precision	recall	f1-score	support
0	0.20	0.33	0.25	3
1	0.00	0.00	0.00	4
accuracy			0.14	7
macro avg	0.10	0.17	0.12	7
weighted avg	0.09	0.14	0.11	7

b)

	precision	recall	f1-score	support
0	0.00	0.00	0.00	2
1	0.33	0.25	0.29	4
accuracy			0.17	6
macro avg	0.17	0.12	0.14	6
weighted avg	0.22	0.17	0.19	6

	precision	recall	f1-score	support
0	0.20	0.50	0.29	2
1	0.00	0.00	0.00	4
accuracy			0.17	6
macro avg	0.10	0.25	0.14	6
weighted avg	0.07	0.17	0.10	6

Classification report of real and synthetic CRC samples of stool, subfigure a) and biopsy samples, subfigure b) of the classifiers Logistic regression (on top) and support vector classifier (On bottom)

	precision	recall	f1-score	support
0	0.20	0.33	0.25	3
1	0.33	0.20	0.25	5
accuracy			0.25	8
macro avg	0.27	0.27	0.25	8
weighted avg	0.28	0.25	0.25	8

	precision	recall	f1-score	support
0	0.25	0.33	0.29	3
1	0.50	0.40	0.44	5
accuracy			0.38	8
macro avg	0.38	0.37	0.37	8
weighted avg	0.41	0.38	0.38	8

Classification report of real and synthetic CRC samples on saliva samples of Logistic Regression (On top) and Support vector classifier (on bottom).

Values of Additional metrics for data evaluation

```
Saliva CRC
Average Similarity Score for Mean Difference: 15.709201164294955
Average Similarity Score for Std Difference: 27.0859916798673
Average Similarity Score for Spearman Correlation: 0.011385215093656 67
Average Similarity Score for Mean Squared Error: 520054.3583764553

Biopsy CRC
Average Similarity Score for Mean Difference: 10.64763885887184
Average Similarity Score for Std Difference: 20.83380992177033
Average Similarity Score for Spearman Correlation: 0.007573280571275771
Average Similarity Score for Mean Squared Error: 256682.8685811541 5

Stool CRC
Average Similarity Score for Mean Difference: 20.277593430534605
Average Similarity Score for Std Difference: 33.864551677857165
Average Similarity Score for Spearman Correlation: 0.052907657717479 87
Average Similarity Score for Mean Squared Error: 363452.0802748448 3

biopsy AP (adenomatous polyps)
Average Similarity Score for Mean Difference: 21.8928285256410 3
Average Similarity Score for Std Difference: 31.3412634415023 1
Average Similarity Score for Spearman Correlation: -0.15461026187268637
Average Similarity Score for Mean Squared Error: 1979617.869350961 9

Average Similarity Score for Mean Difference: 24.25635694866464
Average Similarity Score for Std Difference: 27.57260453758657 8
Average Similarity Score for Spearman Correlation: 0.041640923214943 25
Average Similarity Score for Mean Squared Error: 1783618.389253158 1

Saliva AP
Average Similarity Score for Mean Difference: 29.76647612642906
Average Similarity Score for Std Difference: 26.822502247637 3
Average Similarity Score for Spearman Correlation: -0.16991568296254386
Average Similarity Score for Mean Squared Error: 650798.5956624076

Average Similarity Score for Mean Difference: 30.147304923136794
Average Similarity Score for Std Difference: 56.92598765179215
Average Similarity Score for Spearman Correlation: -0.10809208896012516
Average Similarity Score for Mean Squared Error: 582614.1817474216

Stool AP
Average Similarity Score for Mean Difference: 25.350048309178742
Average Similarity Score for Std Difference: 57.86666557798152
Average Similarity Score for Spearman Correlation: -0.14612709598182608
Average Similarity Score for Mean Squared Error: 541909.1065700484

Average Similarity Score for Mean Difference: 41.936125454197736
Average Similarity Score for Std Difference: 32.44863065720560 5
Average Similarity Score for Spearman Correlation: 0.013504282526331273
Average Similarity Score for Mean Squared Error: 591324.184547714 8
```

ROC curve of Random forest and SVC on the 64-feature dataset

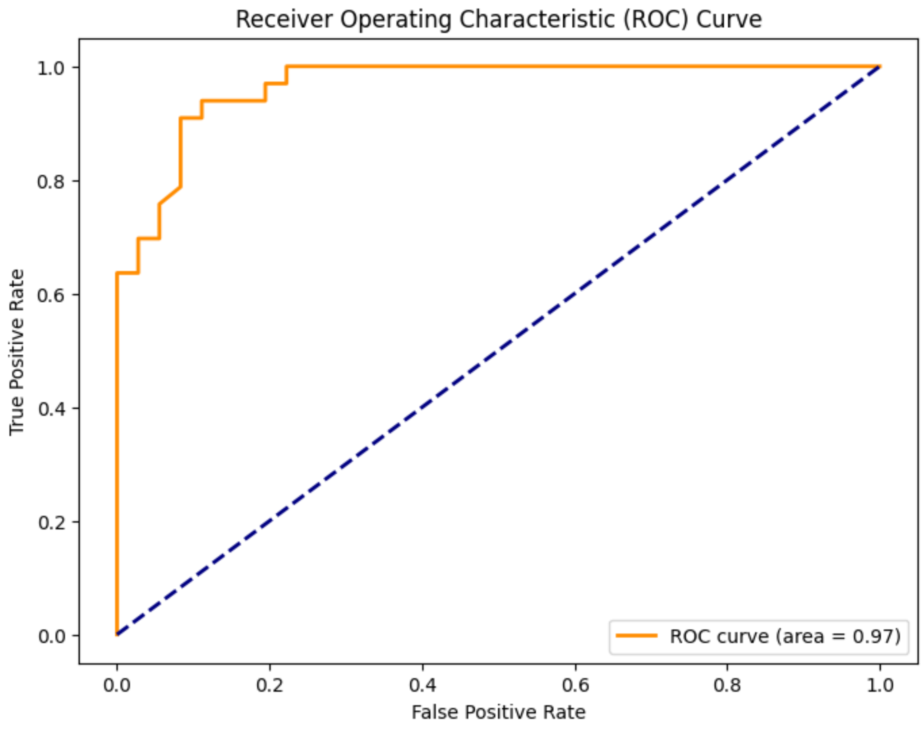

Random Forest classification, accuracy:0.89

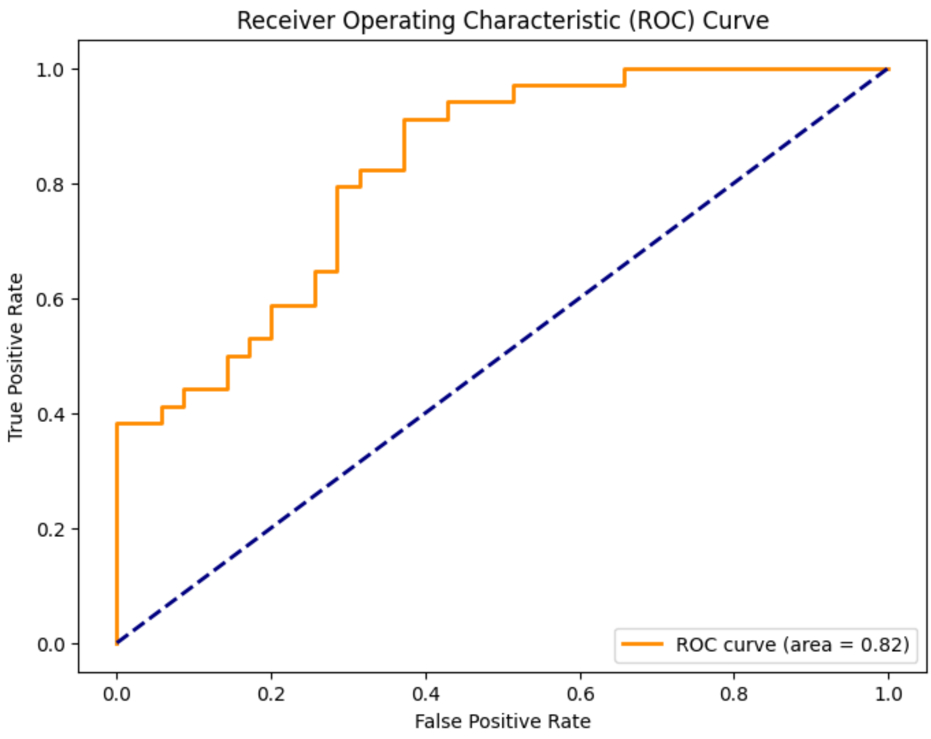

SVC classification, accuracy:0.70

Effect of the two remaining taxa on model predictions in the 64-feature dataset

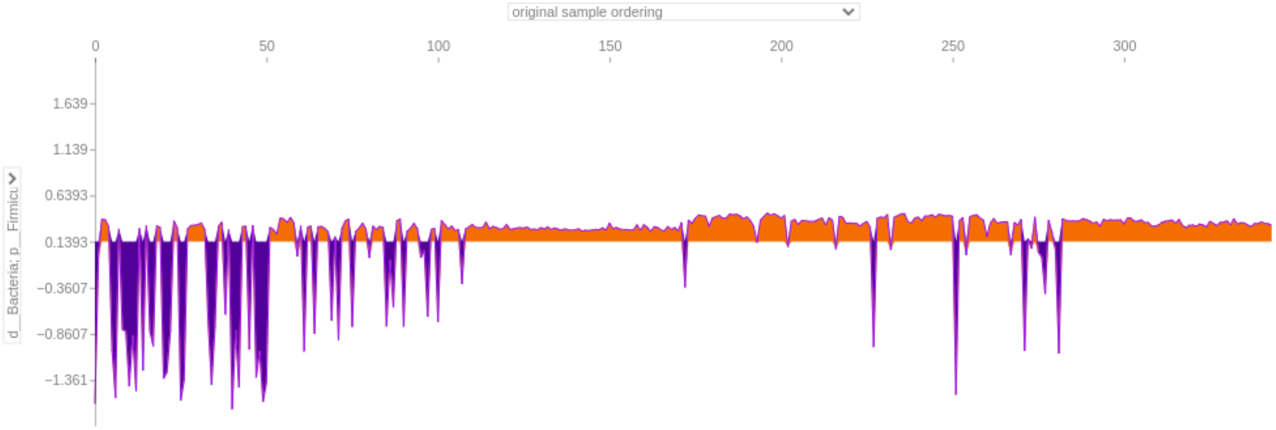

Effect of g. Ruminococcus Gnavus

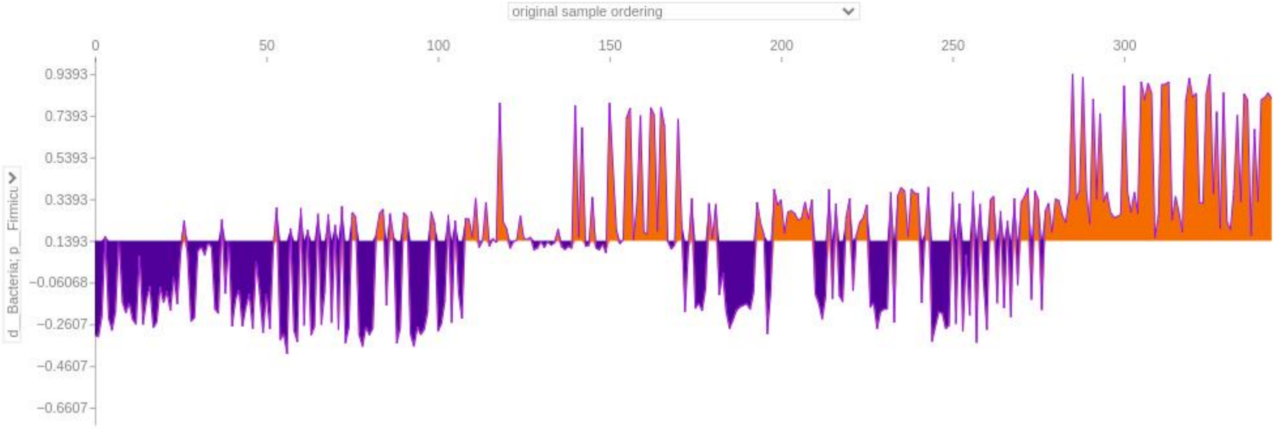

Effect of g. Granulicatella

Effect of taxa on model predictions in the 64-feature stool dataset

The first 53 samples are AP samples, the other 53 are CRC samples

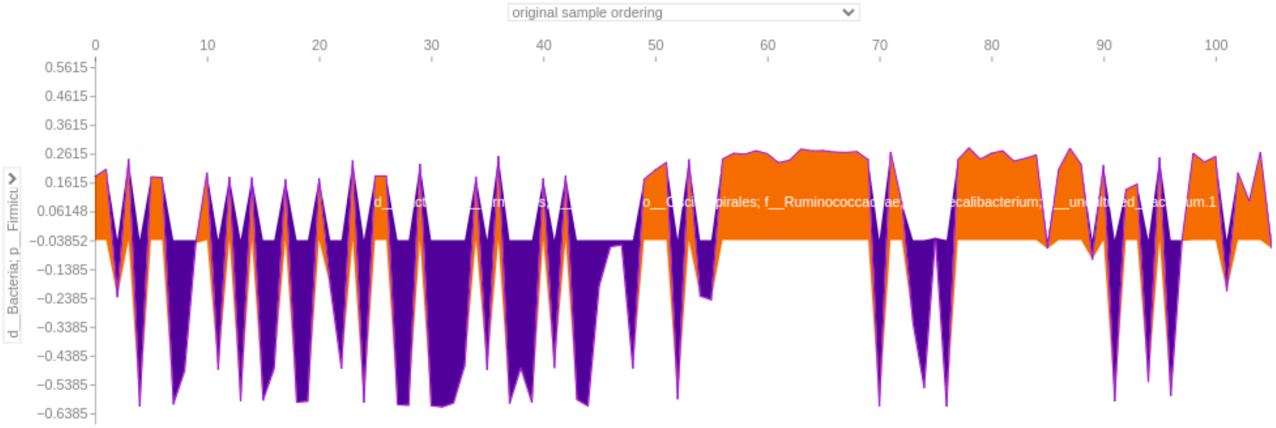

Effect of g. Faecalibacterium

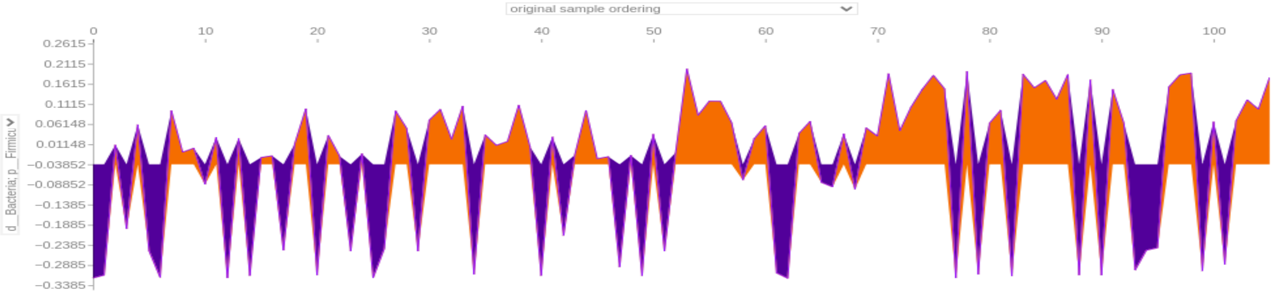

Effect of g. Subduligranulum

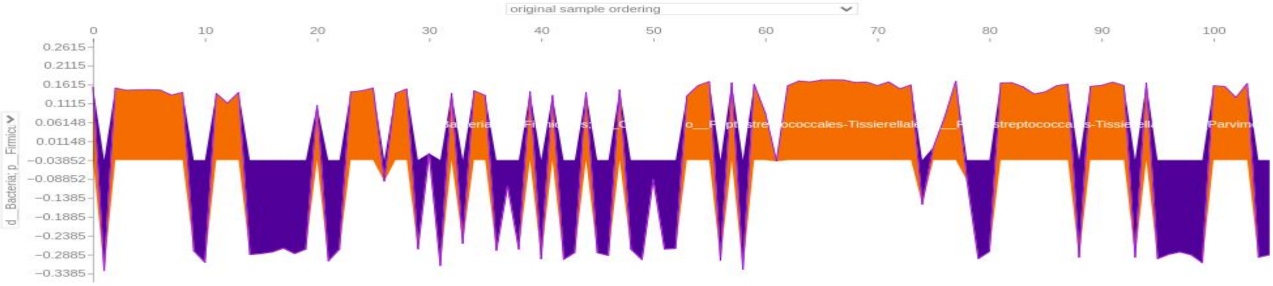

Effect of g.Parvimonas

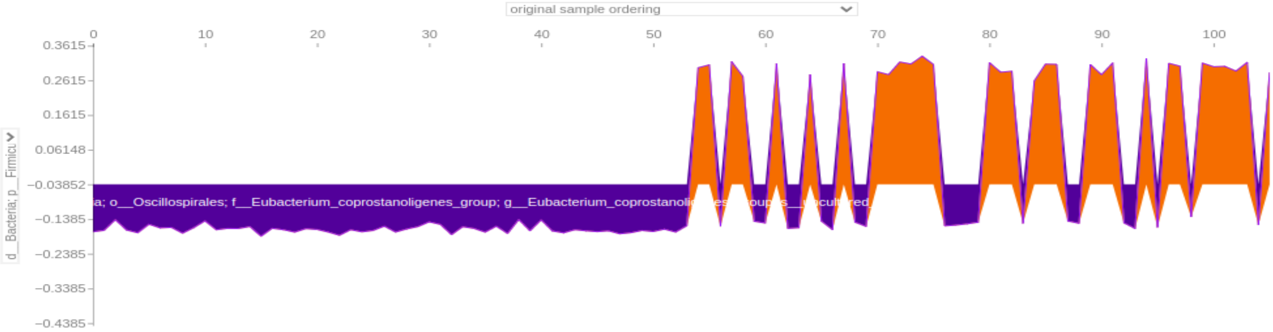

Effect of Eubacterium Coprostanoligenes genus

Effect of taxa on model predictions in the 64-feature biopsy dataset

The first 57 samples are AP samples, the other 57 are CRC samples

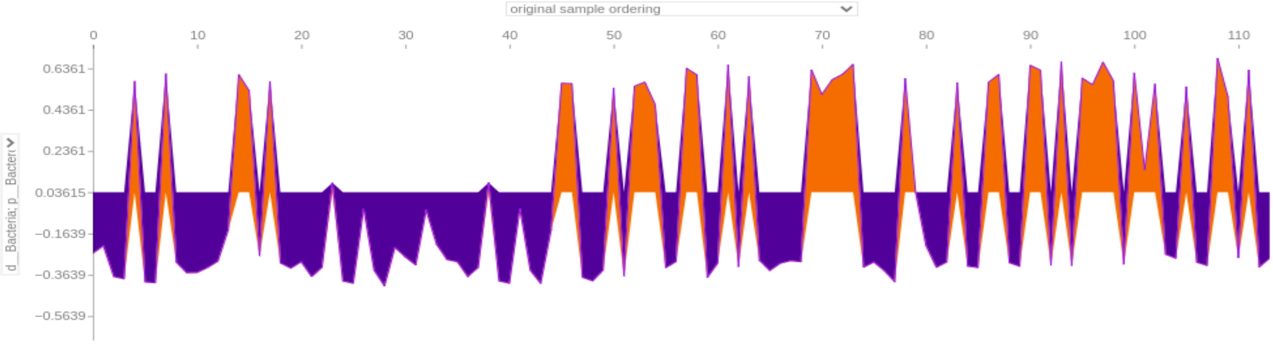

Effect of Alistipes genus

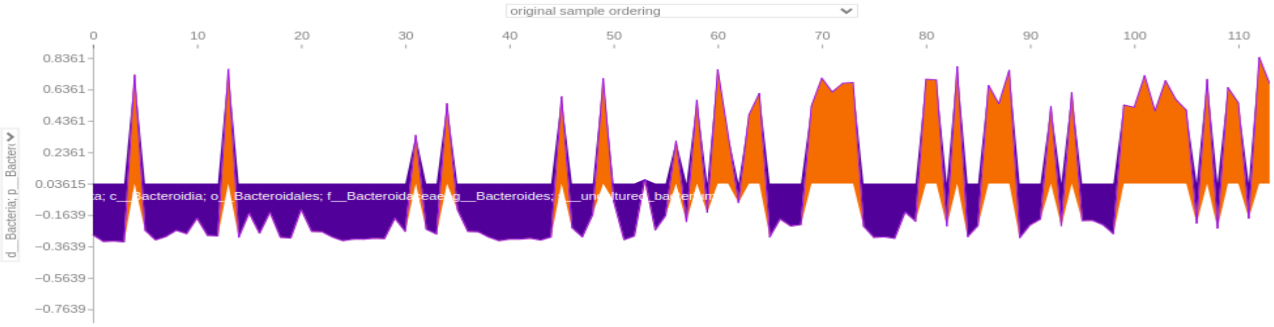

Effect of Bacteroides genus

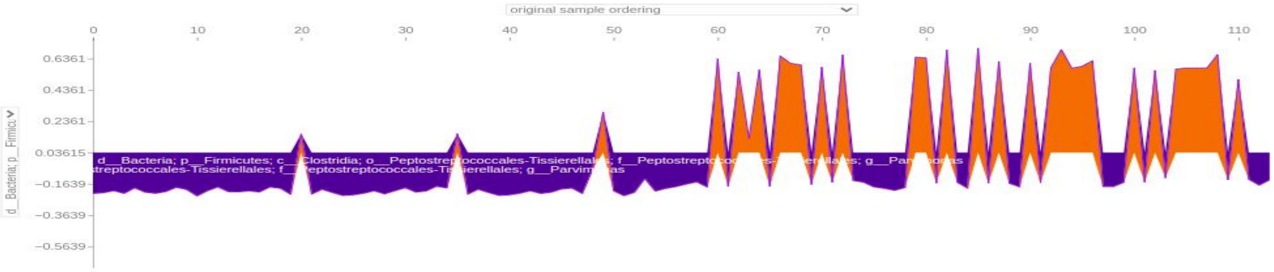

Effect of G. Parvimonas

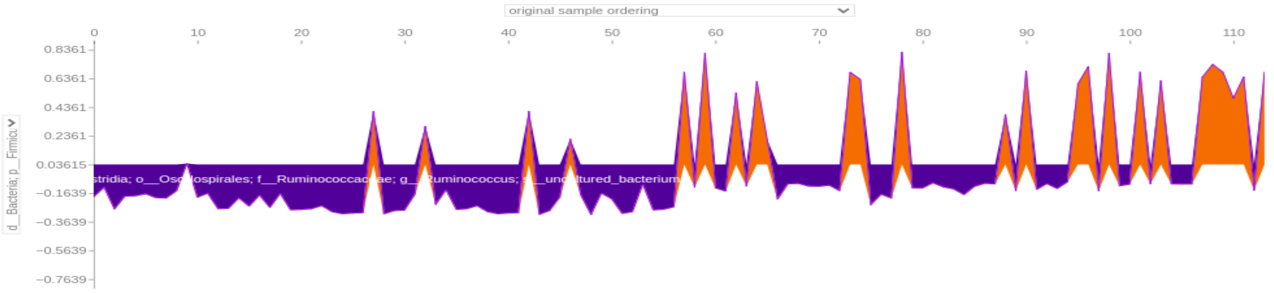

Effect of G.Ruminococcus

Effect of taxa on model predictions in the 64-feature saliva dataset

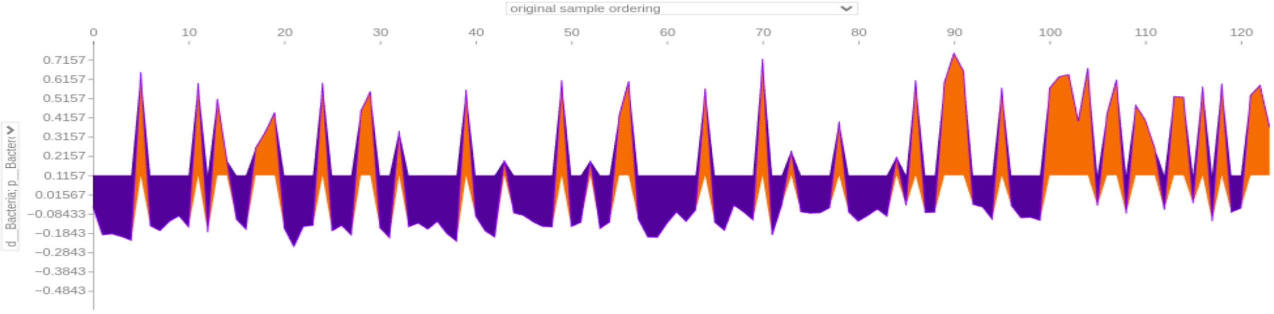

Effect of Prevotella genus

Effect of taxa in common with Russo et al. (2023)
Stool dataset

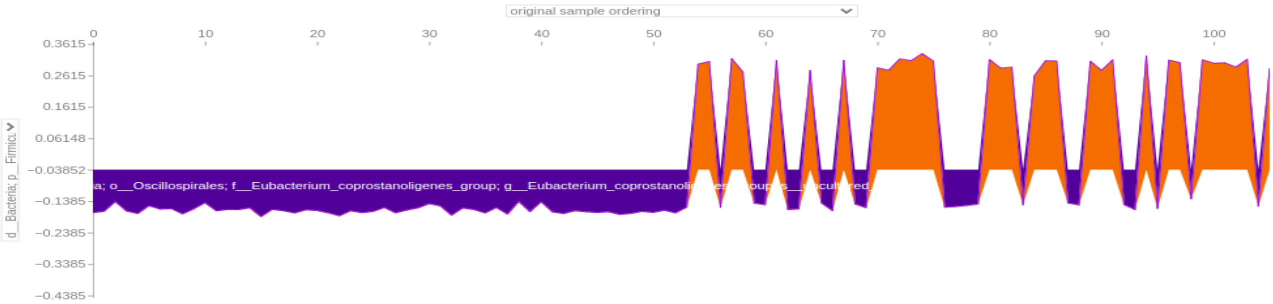

Effect of Eubacterium Coprostanoligenes

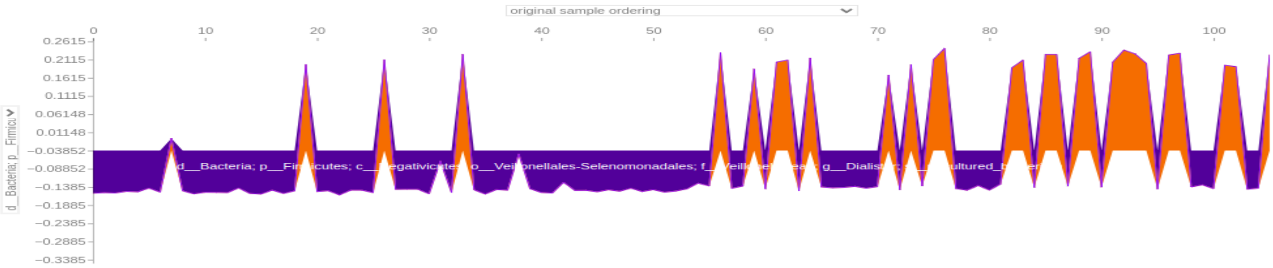

Effect of Dialister genus

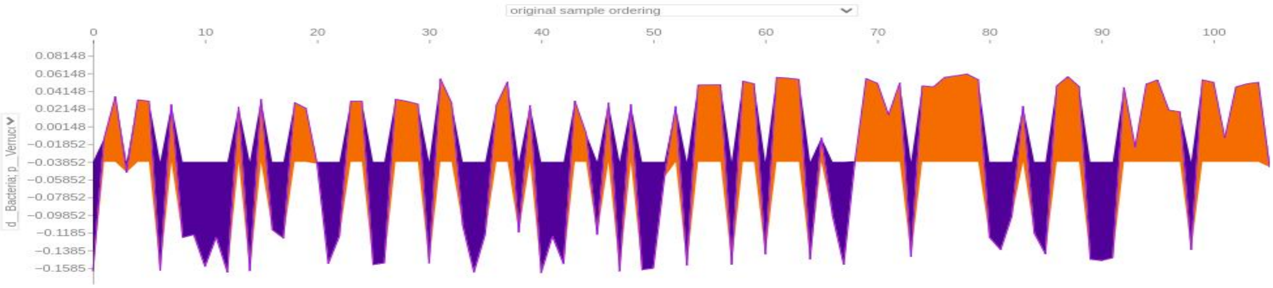

Force plot Akkermnsia

biopsy dataset

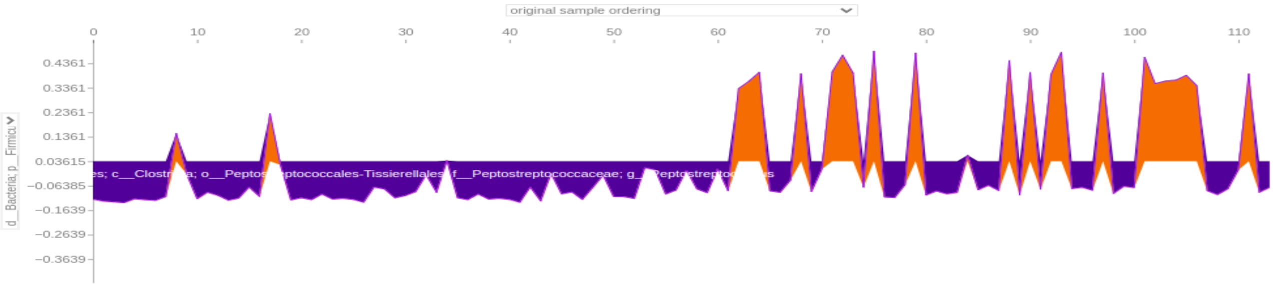

Effect of G.Peptostreptococcus of order Peptostreptococcales/Tisserellales

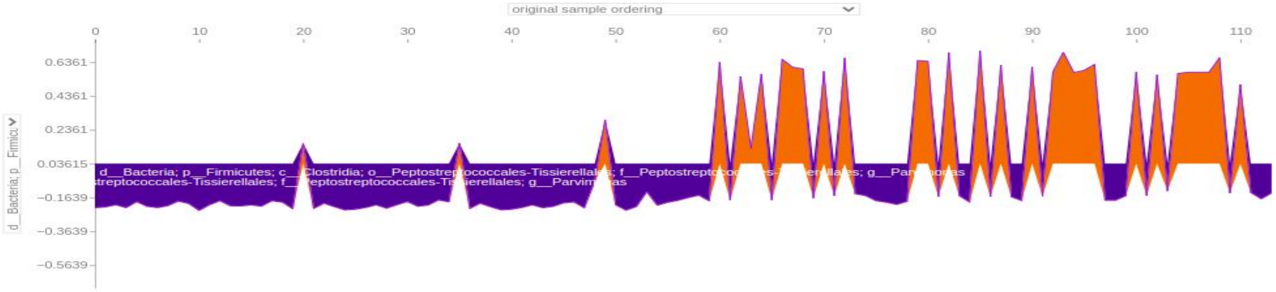

Effect of G.Parvimonas of order Peptostreptococcales/Tisserellales

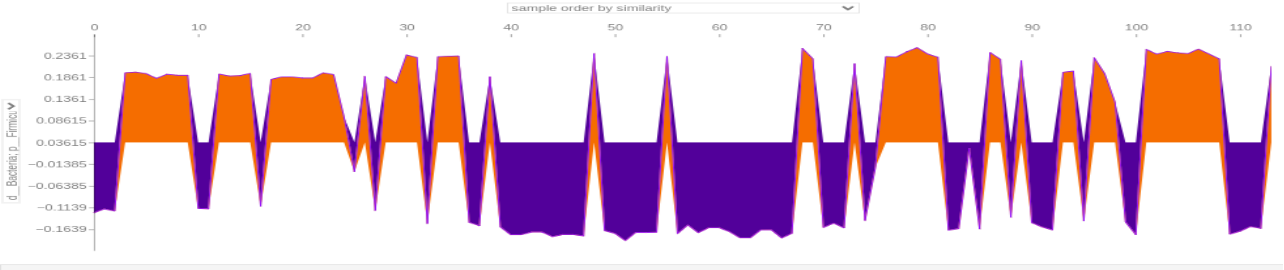

Effect of Granulicatella genus

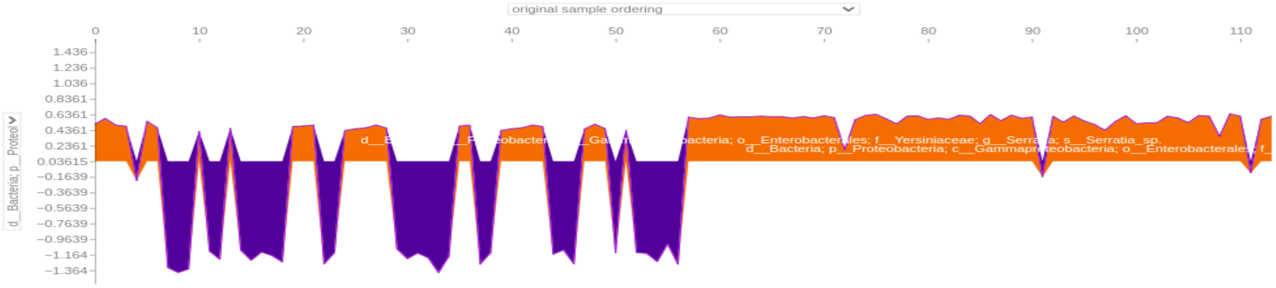

Effect of Serratia genus
